# An assemblage of *Frankia* Cluster II strains from California contains the canonical *nod* genes and also the sulfotransferase gene *nodH*

**DOI:** 10.1186/s12864-016-3140-1

**Published:** 2016-10-12

**Authors:** Thanh Van Nguyen, Daniel Wibberg, Kai Battenberg, Jochen Blom, Brian Vanden Heuvel, Alison M. Berry, Jörn Kalinowski, Katharina Pawlowski

**Affiliations:** 1 Department of Ecology, Environment and Plant Sciences, Stockholm University, 106 91 Stockholm, Sweden; 2 Center for Biotechnology, Bielefeld University, 33615 Bielefeld, Germany; 3 Department of Plant Sciences, University of California Davis, Davis, CA 95616 USA; 4 Bioinformatics and Systems Biology, Justus Liebig University, 35392 Giessen, Germany; 5 Department of Biology, Colorado State University, Pueblo, CO 81001 USA

**Keywords:** *Frankia*, Uncultured, *Datisca glomerata*, *nodABC*, *nodH*, *mce*

## Abstract

**Background:**

The ability to establish root nodule symbioses is restricted to four different plant orders. Soil actinobacteria of the genus *Frankia* can establish a symbiotic relationship with a diverse group of plants within eight different families from three different orders, the Cucurbitales, Fagales and Rosales. Phylogenetically, *Frankia* strains can be divided into four clusters, three of which (I, II, III) contain symbiotic strains. Members of Cluster II nodulate the broadest range of host plants with species from four families from two different orders, growing on six continents. Two Cluster II genomes were sequenced thus far, both from Asia.

**Results:**

In this paper we present the first *Frankia* cluster II genome from North America (California), Dg2, which represents a metagenome of two major and one minor strains. A phylogenetic analysis of the core genomes of 16 *Frankia* strains shows that Cluster II the ancestral group in the genus, also ancestral to the non-symbiotic Cluster IV. Dg2 contains the canonical *nod* genes *nodABC* for the production of lipochitooligosaccharide Nod factors, but also two copies of the sulfotransferase gene *nodH.* In rhizobial systems, sulfation of Nod factors affects their host specificity and their stability.

**Conclusions:**

A comparison with the *nod* gene region of the previously sequenced Dg1 genome from a Cluster II strain from Pakistan shows that the common ancestor of both strains should have contained *nodABC* and *nodH.* Phylogenetically, Dg2 NodH proteins are sister to rhizobial NodH proteins. A *glnA*-based phylogenetic analysis of all Cluster II strains sampled thus far supports the hypothesis that Cluster II *Frankia* strains came to North America with *Datisca glomerata f*ollowing the Madrean-Tethyan pattern.

**Electronic supplementary material:**

The online version of this article (doi:10.1186/s12864-016-3140-1) contains supplementary material, which is available to authorized users.

## Background

Nitrogen is the element that most often limits plant growth. Some prokaryotes can form the enzyme complex nitrogenase to reduce atmospheric dinitrogen and introduce it into the biosphere. Plants can only access this source of nitrogen by entering symbioses with nitrogen-fixing prokaryotes. Root nodule symbioses, where bacteria fix nitrogen while being hosted inside plant cells within special organs, the root nodules, belong to the most efficient of such symbioses. All plants able to enter root nodule symbioses belong to a single dicotyledonous clade, known as the nitrogen-fixing clade made up from the orders Fabales, Fagales, Rosales and Cucurbitales [1, 2]. Two groups of nitrogen-fixing soil bacteria can induce root nodules: rhizobia, a polyphyletic group of proteobacteria, enter symbioses with legumes (Fagales) and with one non-legume genus, *Parasponia* (Cannabaceae, Rosales). Actinobacteria of the genus *Frankia* can induce nodules on a diverse group of plants from 23 genera from eight different families belonging to the orders Fagales (Betulaceae, Casuarinaceae and Myricaceae), Rosales (Elaeagnaceae, Rhamnaceae and Rosaceae) and Cucurbitales (Coriariaceae and Datiscaceae). The distribution of symbiotic species within this clade suggests a common origin of the predisposition to evolve root nodule symbioses assumed to have arisen only once ca. 100 million years ago [1, 2]. Subsequently nitrogen-fixing root nodule symbioses evolved several times independently among the plants with the common predisposition, their independent origins reflected by differences in infection pathways as well as nodule structure and physiology [3].

Based on 16S rDNA phylogeny, the genus *Frankia* has been divided into four clusters, three of which contain symbiotic strains [4]. The non-symbiotic cluster (Cluster IV) represents a heterologous, very diverse group of strains that were isolated from nodules but cannot induce nodule formation. This group is closely related to rhizosphere strains previously detected only by direct amplification of 16S rDNA [5]. The three symbiotic clusters more or less correspond to host-specificity groups. The members of Cluster I can nodulate species of the three actinorhizal families of the order Fagales except for the genus *Gymnostoma (Casuarinaceae)*. Cluster III strains nodulate the actinorhizal Elaeagnaceae (Rosales), and all actinorhizal genera of the Rhamnaceae (Rosales) except for the genus *Ceanothus*, and with the genera *Gymnostoma* (Casuarinaceae) and *Morella* (Myricaceae, Fagales). Cluster II strains form nitrogen-fixing root nodule symbioses with members of the families Coriariaceae and Datiscaceae (Cucurbitales) as well as all the actinorhizal members of the Rosaceae (i.e., the Dryadoideae tribe) and *Ceanothus* (Rosales). In contrast with strains of Clusters I, III and IV, so far only one strain of Cluster II could be cultured, and this strain represents an alkaliphile [6].

Analysis of *Frankia* genomes has shown a surprising variation with regard to genome sizes. Strong differences exist within Cluster I, 5.0 − 5.6 Mb for *Casuarina-*infective strains; [7–10] and ca. 7.5 − 7.7 Mb for the others [7, 11–14]). So far, Cluster II genomes are consistently in the lower size range (5.3–5.6 Mb; [6, 15]). Cluster III genomes range from 7.6 to 10.5 Mb [7, 16–18], similar to Cluster IV genomes (6.9 – 10 Mb; [19, 20]). These size variations have been suggested to reflect differences in saprotrophic potential [4]. Cluster II strains display a large host range and, at least in western North America, low genetic diversity [21] which might indicate a recent evolutionary bottleneck. This is interesting since its host genera from the Cucurbitales, *Datisca* sp. and *Coriaria* sp., show a disjunct distribution [22, 23]. In the case of the genus *Datisca*, the Eurasian species *Datisca cannabina* is found in Pakistan and northwestern India and in the east Mediterranean, while the American species *Datisca glomerata* grows in California and northern Baja California, Mexico [15, 22].

As stated above, *Frankia* Cluster II strains can enter symbioses with members of the Cucurbitales and of the Rosales. The most recent common ancestor of the actinorhizal Cucurbitales, Coriariaceae and Datiscaceae has been molecular-clock dated to 73 +/- 3 mya [24]. Even if we assume that the symbiosis arose not in the common ancestor, but in oldest of both families, the Coriariaceae, the symbiosis would have preceded the diversification of the crown group of *Coriaria*. According to Yokoyama et al. [23], the divergence between the *Coriaria* species from the Northern and the Southern Hemisphere can be dated to 63 or 59 million years ago using fossil-calibrated *rbcL* and *matK* molecular clocks. As for the North American host plants of *Frankia* Cluster II, [25] date the origin of *Ceanothus* in California to 16.6–34.7 mya. The origin of the Dryadoideae tribe of the Rosaceae is dated to the younger Oligocene (23.03–28.1 mya; [26]). These data suggest that North American Cluster II symbioses are significantly younger than the Cucurbitales symbiosis.

*Frankia* Cluster II strains could have reached North America either from South America with *Coriaria ruscifolia* [23, 27], or from Asia when *Datisca* spread from what is now northern India/Pakistan/Nepal to North-East Asia and then to western North America following the Madrean-Tethyan pattern [28]. According to [29, 30] a continuous, sclerophyllous, dry-adapted flora existed between western North America (“Madrean”) and Eurasia (“Tethyan”) across a North Atlantic land bridge during the Ecocene and Oligocene (55 − 25 mya). This is consistent with the timing of *Datisca* speciation: [31] estimated the time of allopatric speciation that led to the distinction between the Eurasian *D. cannabina* and the American *D. glomerata* to 36.5–50.5 mya and 42 mya based on allozyme data and *rbc*L sequences, respectively. In North America, the *Frankia* Cluster II microsymbiont of *Coriaria ruscifolia* or *Datisca* sp. could have extended its host range to *Ceanothus* sp. and the Dryadoideae.

In all phylogenies including different Cluster II strains, strains from New Zealand have always been sister to all other Cluster II strains [16, 21, 32, 33]. Cross-infection studies showing that *Ceanothus* sp. and *Purshia* sp. could not be nodulated in New Zealand [34] suggest strong divergence between *Frankia* Cluster II strains in New Zealand and in North America. There seems to be less divergence between Asian and North American strains, in that a *Frankia* Cluster II strain sampled from soil or nodules of *Coriaria nepalensis* in an area in Pakistan where both *C. nepalensis* and *D. cannabina* are distributed, can nodulate the Californian *Datisca* species *D. glomerata* [15]. This makes it more likely that Cluster II *Frankia* strains in North America originated from nodules of *Datisca* sp. which arrived from Asia *via* the Bering Strait.

The first genome of a *Frankia* Cluster II strain to be sequenced, *Candidatus* Frankia datiscae Dg1, originated from nodules of *C. nepalensis* in Pakistan [15, 35]. The second sequenced genome of a Cluster II strain, BMG5.1, originated from nodules of *Coriaria japonica* growing in Japan [6]. Thus, the analysis of a *Frankia* Cluster II genome from an area devoid of host plants from the genus *Coriaria* could promise novel insights into the diversity within Cluster II and the differences between the three symbiotic *Frankia* clusters. Specifically, the genome of a North American strain promises to reveal information on the evolution and distribution of *Frankia* Cluster II from Asia/New Zealand/South America to North America.

The evolution of root nodule symbioses involved the recruitment of mechanisms from the evolutionarily older arbuscular mycorrhizal (AM) symbioses [36]. In legume/rhizobia symbioses, flavonoids in the root exudates of the host plants induce the expression of bacterial nodulation (*nod*) genes leading to the synthesis of lipochitooligosaccharide (LCO) signal molecules, the Nod factors. Also AM fungi produce LCO signal factors [37]. These LCOs are perceived by plant kinases of the LysM family and activate the common symbiotic pathway (CSP) that controls both legume/rhizobia and AM symbioses ([38]; reviewed by [39]). This CSP is also used for microsymbiont signaling in actinorhizal symbioses, as shown for *D. glomerata* [40] and *Casuarina glauca* [41, 42]. In this context, it seems likely that symbiotic signaling of *Frankia* strains also involved LCO-like compounds that are perceived by LysM receptor kinases. However, so far only the genome of *Candidatus* Frankia datiscae Dg1 has been shown to contain the canonical *nod* genes *nodABC* which in rhizobia are responsible for the synthesis of the common backbone of the LCO Nod factors [15, 35]. The second Cluster II strain to be sequenced, BMG5.1 did not contain homologs of the canonical *nod* genes in the draft genome [6].

In order to gain more insight into the evolution of North American Cluster II *Frankia* strains and to answer the question whether the presence of the canonical *nod* genes in Dg1 typical for Cluster II, or represents an exception, we sequenced the genome of a Cluster II inoculum from *D. glomerata* in California.

## Methods

### Plant and bacterial material

*Datisca glomerata* (C. Presl) Baill. seeds originating from plants growing at Gates Canyon in Vacaville, California, USA. Plant seedlings were grown under sterile condition to avoid contamination of *Frankia* strain Dg1 which were growing in the greenhouse. Seedlings were then transferred to pot filled with autoclaved mixture of soil and sand, ratio 1:1. Plants were grown in a growth chamber under a 15 h/9 h light dark cycle and day/night temperature of 23 °C/19 °C, relative humidity 65 % and light intensity of 60–100 μEm^-2^s^-1^. A soil sample containing the uncultured *Frankia* strain was acquired under a growing *D. glomerata* at Gates Canyon, Vacaville, California. Nine-week old *D. glomerata* plants in the growth chamber were inoculated with this infected soil. Inoculated plants were fertilized with 1-quarter-strength Hoagland’s medium without nitrogen [43].

### Isolation of genomic DNA from isolated vesicle clusters

To isolate the *Frankia* vesicle clusters from root nodules, the protocol from Lundquist and Huss-Danell [44] was applied with some modifications. Root nodules harvested from *D. glomerata* were surfaced sterilized in 1 % sodium hypochlorite for 10 min. Then they were washed with sterile dd H_2_O. Nodules were homogenized in a mortar in homogenization buffer (0.05 M Tris-HCl pH 7.9–8, 4 % (w/v) PVP, 0.1 M KCl, 5 mM EDTA, 0.6 M sucrose, 10 mM Na_2_S_2_O_4_). The homogenized tissue was poured over an 80 μm nylon filter and the flow through was poured over a 16 μm nylon filter. The 16 μm filter was washed several times with a wash buffer (50 mM HEPES pH 7.8, 4 % (w/v) PVP, 10 mM EGTA, 10 mM EDTA, 2 mM Na_2_S_2_O_4_) and the cells obtained on the filter were transferred to a new tube containing pectinase buffer (10 mM Tris-HCl pH 6, 10 mM NaCl). The cells were incubated with pectinase (Macerozyme R10; Saveen-Werner, Sweden) at 37 °C for 2 h, shaking slowly (110 rpm). The vesicle clusters were spun down and resuspend in TES buffer (0.3 M sucrose, 25 mM EDTA pH 8.0, 25 mM Tris/HCl pH 8.0). The vesicle clusters were broken using the ultrasonic homogenizer Sonoplus HD 2070 (Bandelin Electronic) at 90 % amplitude and 30 % pulsing for 30 s. The genomic DNA then was extracted using CN solution (10 % CTAB in 0.7 M NaCl) and phenol/chloroform (1:1).

### Sequencing and genome assembly of the *Candidatus* Frankia datiscae Dg2 metagenome

The quality of the DNA was assessed by gel-electrophoresis and the quantity was estimated using the Quant-iT PicoGreen dsDNA kit (Invitrogen) and the Tecan Infinite 200 Microplate Reader (Tecan Deutschland GmbH, Mainz, Germany). To obtain the complete genome sequence, a whole-genome-shotgun PCRfree (Nextera DNA Sample Prep Kit; Illumina, Munich, Germany) and an 8 K mate pair library (Nextera Mate Pair Sample Preparation Kit; Illumina) were generated based on the manufacturer’s protocol. After sequencing and processing of the raw data, a *de novo* assembly was performed using the GS *De Novo* Assembler software release version 2.8 (Roche, Mannheim, Germany) with default settings. In our approach, we used a 2 x 300 bp paired end sequencing run. For quality-control and filtering, a pipeline including trimmomatic (Bolger et al. [45]), r2cat [46] and contig-length *vs.* read-count analysis [47, 48] was implemented. The resulting data set was manually inspected and improved.

### Reference assembly on the *Candidatus* F. datiscae Dg2 metagenome from the metagenome dataset

Based on contig length vs. read count analysis [47–49] and on taxonomic profiling by applying LCA [50], the established dataset was classified as a metagenome dataset including different *Frankia* strains, *Datisca glomerata* and a low amount of a diverse bacterial strains. To identify related species of *Frankia* in the metagenome dataset, a reference assembly was applied. For reference assembly, metagenomic contigs were mapped onto the reference genome *Frankia* sp. Dg1 [15] by means of *r2cat* [46]*.* Mapped contigs and their corresponding reads were extracted and *de novo* assembled using the GS Assembler (version 2.8; Roche) with default settings. Furthermore, automatic gene prediction and annotation of coding sequences on metagenomic *Frankia* contigs was performed within the genome annotation system GenDB 2.0 [51] as described previously [47, 52, 53]. An *in silico* gap closure approach was performed to close the gap between the two *nodC* contigs [49, 52, 54, 55].

Nucleotide sequence data for the reconstructed *Frankia* sp. Dg2 strains were deposited in the EBI database (accession numbers FLUV01000001-FLUV01002738). The *nod* region was finished manually and therefore separately deposited at EMBL (accession number LT622247).

### Identification of single-nucleotide polymorphisms in the Dg2 metagenome

SNP and DIP detection is 1 of the key analyses to estimate the amount of included strains in a reconstructed reference sequence out of a metagenome. Therefore metagenomic reads were mapped on the reconstructed reference sequence of *Frankia* sp. Dg2 by applying gsMapper (Roche) using strict settings (90 % sequence, 100 bp minimal overlap). SNP and DIP detection was performed by means of ReadXplorer [56]. The implemented SNP and DIP detection in ReadXplorer not only reveals small-scale evolutionary differences, but also allows analysis of the resulting functional differences emerging from polymorphisms. ReadXplorer detects SNPs and DIPs based on a user-definable minimum percentage of variation and a minimum count of mismatching bases in the mappings at the examined position. This allows the detection of small amounts of underrepresented strains in a reconstructed reference sequence.

### Phylogenetic analysis and comparison of the reconstructed Dg2 metagenome to the genomes of completely sequenced and annotated members of the genus *Frankia*

To compare and phylogenetically classify *Frankia* sp. Dg2 in relation to completely sequenced and annotated members of the genus *Frankia*, the comparative genomics platform EDGAR was applied [57]. The core genome of all selected strains was calculated by means of EDGAR and based on all core genes for each *Frankia* strain, phylogenetic distances were calculated from multiple sequence alignments. A phylogenetic tree was deduced from concatenated core gene alignments using PHYLIP [58].

### Identification of *Frankia* genes involved in secondary metabolite synthesis by applying antiSMASH

antiSMASH [59, 60] is the first comprehensive pipeline enabling identification of biosynthetic loci covering the whole range of known secondary metabolite compound classes. This tool was used to search for secondary metabolite synthesis clusters in the different available *Frankia* genomes. For this approach, all *Frankia* genomes were selected and imported in antiSMASH. The output was in detail manually analyzed for secondary metabolite synthesis clusters and compared with related clusters in our bacteria.

### Read-based comparative analysis based on fragment recruitment

Fragment recruitment approaches were performed as described previously [61]. Briefly, reads that led to low abundant SNPs to the Dg2 metagenome sequence were exported and aligned to *Frankia* sp. Dg1 genome and Dg2 metagenome sequences by applying blastN [62]. Reads were used for further evaluation if at least 80 % of a read were aligned to one of the target sequences and if the identity was at least 55 %. Sequence homology to Dg1 and Dg2 was compared to get better insight into the taxonomical relationship of this low abundant *Frankia* strain.

### Average nucleotide identity and genome alignment of *Frankia* Cluster II strains

Average nucleotide identity (ANI) was analysed as described previously [63, 64, 65] to determine the relationship between different *Frankia* genomes.

MAUVE [66] was used to align and to visualize rearrangements in the chromosomes of *Candidatus* Frankia datiscae Dg1 *vs.* *Frankia* sp. Dg2 and *Frankia* sp. BMG5.1, *Frankia alni* ACN14a vs. *Frankia* sp. ACN1^ag^, *Frankia alni* ACN14a vs. *Frankia* sp. QA3 and in *Frankia * sp. CcI3 vs. *Frankia * sp. BMG5.23, respectively. The bioinformatics tool MAUVE including the progressiveMauve algorithm was applied with default settings for the comparative analysis [67]. Before applying Mauve, r2cat  [46] was used to adapt order and direction of contings.

### Protein phylogeny

The sequences encoded by the open reading frames identified as putative *nodH* genes from the assembled Dg2 metagenome (*Dg2nodH1*, FDG2_3270; *Dg2nodH2,* FDG2_3293) were each used as a query for a separate BLASTX search against two databases, the non-redundant (nr) dataset of GenBank (searched on 2015.12.17), and all genomes available in JGI-IMG (searched on 2015.12.17). A total of 204 amino acid sequences from ten different genera were identified with similarity of 1e^-20^ or above to at least one of the two putative orthologs. A total of 25 sequences were collected by selecting, from each genus, up to three sequences from unique species with the best e-values. These sequences were combined with Dg2nodH1 and Dg2nodH2 to generate a dendrogram.

The collected amino acid sequences were first aligned using MAFFT [68]. Aligned sequences were then analyzed using ProtTest3 [69] to estimate the best amino acid substitution model. The estimated parameters were then used in GARLI 2.0 [70] under default settings to generate a maximum likelihood tree. Three parallel searches were conducted in order to avoid selecting a tree lodged on a local optimum. Bootstrap analysis was conducted with 100 replicates. For each bootstrap replicate, parameters were estimated by GARLI 2.0 and two parallel searches were conducted.

### GlnA-based phylogeny of *Frankia* strains

Field samples were collected and specimen vouchers were deposited in the herbarium of UC Davis (CA, USA). DNA was extracted from single nodule lobes using a modified CTAB procedure [71] after the nodule lobes had been washed with ddH_2_O, TEA buffer, and the periderm had been removed. PCR of a partial sequence of the *glnA* gene using primers *DB41* and *DB44* [33] was conducted. Fifty microliter PCR reactions (10 ng template DNA, each primer at 0.5 μM, 2 mM MgCl_2_, 1 unit of *Taq* DNA polymerase, 20 μM of each dNTP, and 5 μl of 10X buffer) were run on thermocycler (Perkin-Elmer manual, Perkin-Elmer Corp., Norwalk, CT) programmed for a hot start (95 ° C, 2 min.) and 30 cycles of 94 ° C for 30 s, 57 ° C for 45 s, and 72 ° C for 1 min with a final extension for 7 min. A negative control, lacking template DNA, was also run. PCR amplifications were analyzed in a 1.5 % agarose gel run in 0.5 X TBE buffer and visualized with ethidium bromide under UV light. PCR products were cloned using a TOPO TA cloning kit (Invitrogen) and sequenced using Big Dye reagents (Applied Biosystems, Foster City, CA) and analyzed on an ABI Prism 3100 automated sequencer (Applied Biosystems) according to the manufacturers’ instructions.

Sequences were proofread and assembled using SeqMan (1998, DNAstar, Madison, WI.) and then Blast searched at the NCBI Home page (www.ncbi.nlm.nih.gov/BLAST/) to identify and categorize sequences. Cluster II *Frankia* sequences generated were then aligned with existing Cluster II *Frankia glnA* sequences.

All alignments were created using MUSCLE (multiple sequence comparison by log-expectation [72]; Edgar 2004) at the EMBL-EBI website. Maximum Likelihood analyses were performed using PhyML 3.0 [73] on the ATGC bioinformatics server. The substitution model was GTR, with estimated invariant sites, and rate variation sampled from a gamma distribution. Support for branches was evaluated using bootstrap analysis as above.

### Real time quantitative reverse transcription PCR (RT-qPCR) for the analysis of gene expression in nodules

RNA was isolated as described by Persson et al. [35], but using the Spectrum Plant Total RNA Kit (Sigma-Aldrich, Stockholm Sweden) from young *D. glomerata* nodules (i.e., nodules with two to three lobes) harvested eight weeks after infection. RT-qPCR analysis was performed as described by Persson et al. [35]. Reverse transcription was done using the SuperScript IV First Strand Synthesis System (Invitrogen). Primers were designed using Primer3Plus [74].

## Results

### Sequencing of the *Candidatus* Frankia datiscae Dg2 metagenome

DNA isolated from vesicle clusters originating from a *Datisca glomerata* plant growing at Gates Canyon, Vacaville, CA, USA, was *de novo* sequenced by applying a strategy combining whole-genome-shotgun and mate pair sequencing on the Illumina MiSeq platform. Sequencing on MiSeq platform resulted in 8,217,565 reads with a total of about 2.1 Gb sequence information. Assembly of the data by applying of the gsAssembler 2.8 resulted in 52,586 contigs, 1441 scaffolds and a size of 28.3 Mb. First, all assembled reads were taxonomically classified by applying LCA [50]. In total, about 1,700,000 reads were classified to the domain *Bacteria.* Mainly these reads were classified to the genus *Frankia* (ca. 1,150,000 reads), but also to *Streptomyces* (ca. 28,000 reads) and *Mycobacterium* (ca. 4,534 reads). In addition, ca. 210,000 reads were classified to *Eukaryota.* To gain deeper insights into the sample composition, a ’contig-length *vs*. read-count‘plot was calculated (Additional file 1; [47–49]). Obtained results represented a point cloud, but two contig groups at 1x and at 10x were apparent. Contigs of both groups were analyzed by means BLASTn [62] to deduce further taxonomical information on these contigs. The underrepresented contigs of the 1x group were assigned to *Datisca glomerata* and to diverse bacterial strains, whereas the other group represents contigs belonging to *Frankia*. The data were filtered by means of a reference mapping to *Frankia* sp. Dg1 applying *r2cat.* This mapping approach was aimed to reconstruct the *Frankia* genome from the identified metagenome. In total, 3,269,398 reads (40.68 % of all metagenome sequence reads) amounting to 576.12 Mb sequence information were extracted for further analysis. Further genome assembly of extracted reads related to the *Frankia* Dg1 genome applying the GS assembler (version 2.8) yielded 8,283 contigs (1066 scaffolds) and amounting a total length of 5.92 Mb.

### Detection of different *Frankia* strains in a reconstructed draft genome by means of SNP and sequence analysis

The relatively high number of assembled contigs and scaffolds suggested that the sample contains more than one *Frankia* strain. To determine the degree of conservation of the *Frankia* sp. Dg2 sequence, all sequence reads of the project were mapped on the reconstructed Dg2 metagenome. The mapping approach was aimed at the estimation of *Frankia* species in the metagenome. In total, 6,234,666 reads (75.87 % of all metagenome sequence reads) yielding approximately 1.59 Gb sequence information were mapped on the Dg2 metagenome sequence. Based on SNP calling by applying ReadXplorer [56], 158,053 SNPs were predicted. More than 80 % of the SNPs show a low coverage <10 % of all bases at a certain variable position. The low abundant SNPs were always represented by a particular base and no additional variants were found. That may indicate a low abundant, related *Frankia* strain in the metagenomic sample.

To get taxonomical information about this strain, 44,620 reads leading to the low abundant SNPs were exported and used for a fragment recruitment using Dg1 and Dg2 as reference sequence. In both fragment recruitment approaches, 99 % of all reads were mapped to the reference sequences. Mapped reads to the reference genomes showed an average sequence identity of 96.6 % to Dg1, whereas the average sequence identity to Dg2 was somewhat lower (93.8 %). Therefore this putative strain is more closely related to Dg1.

In combination with the SNP approach, a visual inspection of the assembly data was performed. In a few cases, a 50−50 distribution of SNPs in comparison to the consensus sequence was observed. In addition, conserved *Frankia* contigs are followed by two different contig variants, detected in 267 cases. Local similarities were detected frequently within such contig pairs. It is very likely that these contig pairs contain variants of *Frankia* genes/regions that are significantly different to each other and therefore were not assembled into one contig. Occurrence of sequence differences is not evenly distributed over the aligned and matching contig segments indicating that some regions are more conserved than others, e.g. regions with housekeeping genes are highly conserved in both *Frankia* strains. Therefore, such local similarities between contigs in first instance confirm the existence of two closely related *Frankia* sp. Dg2 strains. These genomes share conserved regions without a SNP, but also contain some individual regions. In total, 198 of 267 contig variants represent variations in intergenic regions, whereas the remaining variants mainly refer to insertions/deletions of mobile genetic elements or changes in genes encoding hypothetical proteins.

The reconstructed *Frankia* sp. Dg2 metagenome contains sequence information of three different *Frankia* strains originating from *D. glomerata* nodules. Based on a SNP calling approach in ReadXplorer [56] including a manual inspection of the assembly and mapping result, these three different *Frankia* strains were detected. With bioinformatic methods, it was not possible to split the two abundant *Frankia* sp. Dg2 strains, Dg2a and Dg2b, in separate datasets because of their close relatedness (ca. 98–99 %) and a similar abundance in the sample (ca. 55 % Dg2a, ca. 45 % Dg2b). Therefore, the reconstructed sequence will be called “metagenome” in the next chapters, because it contains sequence information of 2 closely related *Frankia* strains. The third strain, Dg2c, was so underrepresented in the sample (ca. 1 %) that its effect on the consensus sequence could be ignored.

### Features of the reconstructed Dg2 metagenome

The final sequence of the *Frankia* sp. Dg2 metagenome was established as described above. Based on scaffold information it could be proven that the *Frankia* sp. Dg2 metagenome only comprises a circular chromosome. Additional *Frankia* plasmids were not detected in the remaining metagenome data. Considering the final size of the *Frankia* sp. Dg2 metagenome, a 93-fold coverage was achieved after different filter steps. Relevant data of the *Frankia* sp. Dg2 metagenome project are summarized in Table 1. The circular chromosome of the *Frankia* sp. Dg2 metagenome has a size of 5,929,312 bp and features a GC content of 67.90 %. Gene prediction and annotation of the assembled metagenome sequence were performed within the GenDB system [51]. This approach resulted in the prediction of 6,536 coding sequences, 36 tRNA genes, and two *rrn* operons. The *Frankia* sp. Dg2 metagenome sequence contained a typical origin of replication *oriC*. Housekeeping genes, e.g. genes involved in glycolysis, essential for the survival of the bacterium, were identified on the circular chromosome of *Frankia* sp. Dg2. Due to this fact, the reconstructed *Frankia* sp. Dg2 sequence represents a characteristic first chromosome. In addition, a region related to the plasmid pFSYMDG01 of Dg1 was found on a contig originating from the Dg2 chromosome. It seems that *Frankia* plasmids can integrate into the chromosome, which is not surprising because the *parBA* gene system, encoding a chromosome/plasmid partitioning system, was found in both the Dg1 chromosome (FsymDg_4545 and FsymDg_4546) and in the Dg2 metagenome (FDG2_6558 and DG2_6557; *parA/parB*).

**Table 1:** Sequencing and assembly statistics of the reconstructed *Candidatus* F. datiscae Dg2 metagenome

Features	*Frankia* sp. Dg2
All Reads	8,217,565
All Bases	2,163,821,348
Filtered Reads for genome reconstruction	3,269,398
Filtered Bases for genome reconstruction	576,128,786
Assembled Reads for genome reconstruction	3,123,854
Assembled Bases for genome reconstruction	550,550,122
Contigs	8283
Largest Contig	36,613 bp
Scaffolds	1066
Largest Scaffold	368,548 bp
Reconstructed genome size	5.929.312 bp
Predicted genes	6536
Genes with predicted function	3248
tRNA	36
*rrn*-Operons	2
GC content	67.90 %

### Core genome comparison of Dg2 with other *Frankia* genomes

**Fig. 1:**
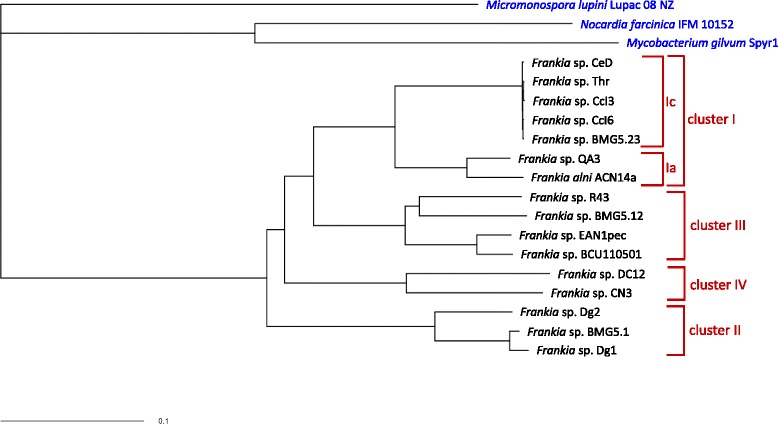
Comparison of core genomes of 16 sequenced *Frankia* strains [5, 7–11, 14–20, 94] using EDGAR [57]. Outgroups (given in blue) were three actinobacterial genomes, *Micromonospora lupini* Lupac 08 [95], *Nocardia farcinica* [96] and *Mycobacterium gilvum* Spyr1 [97]. The phylogenetic tree was deduced from concatenated core gene alignments using PHYLIP [58]. Bootstrap values were 100 for every branch

Using EDGAR [57] a phylogenetic tree of *Frankia* strains was calculated using core genome comparison (Fig. 1). In this tree, Cluster II is the basal symbiotic *Frankia* cluster as previously described [6, 35, 75]. Consistent with the data presented by Gtari et al. [6], Cluster II is not only sister to the other symbiotic Clusters I and III, but also to the non-symbiotic Cluster IV.

Within Cluster II, Dg2 (California) is sister to Dg1 (Pakistan; [15]) and BMG5.1 (Japan; [6]). However, given that this phylogeny does not contain a strain from New Zealand which are the ancestral strains in Cluster II in all other phylogenies, this cannot be interpreted to mean that North American Cluster II strains are ancestral to Eurasian ones. At any rate, the comparison shows that in contrast with North American Cluster II *Frankia* strains [21], Eurasian ones do not show low genetic diversity.

### Average nucleotide identity (ANI) and alignment of genomes of *Frankia* cluster II strains

To determine similarities between the different *Frankia* genomes of the Clusters I and II, pairwise Average Nucleotide Identies (ANI) were calculated. Usually genomes of prokaryotic isolates, which belong to the same species, have ANI values above 95 % [63].

**Fig. 2:**
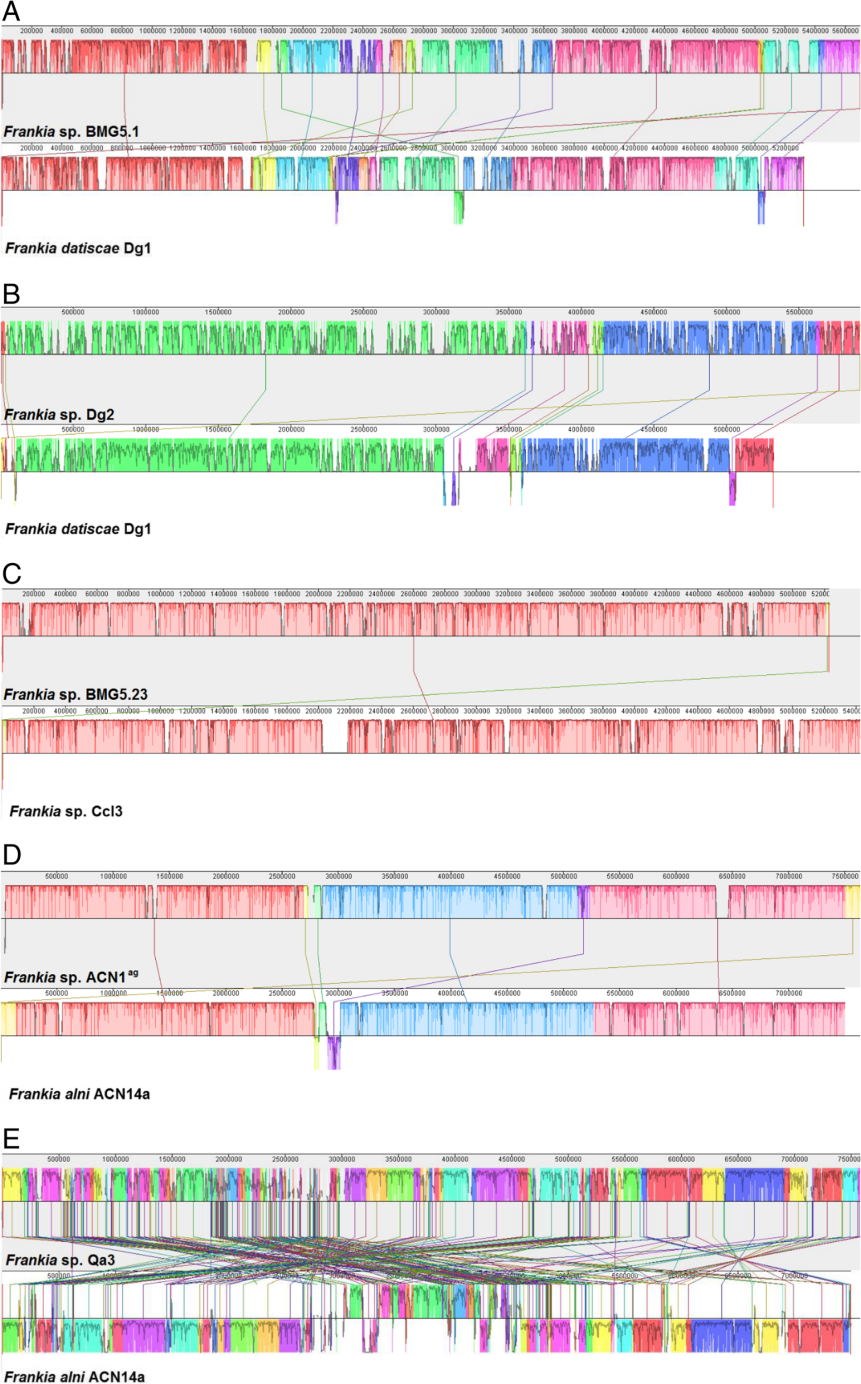
*Frankia* genome alignments using MAUVE [66]. Chromosome coordinates are plotted on the x-axis and the y-axis denotes the percentage of sequence identity. Colored areas appear above and possibly below the center line. Each of these areas surrounds a region of the genome sequence that aligned to part of another genome, and is presumably homologous and internally free from genomic rearrangement. When an area lies above the center line the aligned region is in the forward orientation relative to the first genome sequence. Areas below the center line indicate regions that align in the reverse complement orientation. Regions outside the colored areas lack detectable homology among the input genomes. Inside each area, Mauve draws a similarity profile of the genome sequence. The height of the similarity profile corresponds to the average level of conservation in that region of the genome sequence. Regions that are completely white were not aligned and probably contain sequence elements specific to a particular genome. The height of the similarity profile is calculated to be inversely proportional to the average alignment column entropy over a region of the alignment. **a** Pairwise alignment of the genomes of *Candidatus* Frankia datiscae Dg1 (chromosome) *and Candidatus* Frankia datiscae Dg2 (1066 scaffolds) (88 % ANI). **b** Pairwise alignment of the genomes of *Candidatus* Frankia datiscae Dg1 (chromosome) and *Frankia* sp. BMG5.1 (116 scaffolds) (96 % ANI). **c** Pairwise alignment of the genomes of *Frankia* sp. CcI3 (chromosome) and *Frankia* sp. BMG5.23 (166 scaffolds) (99 % ANI). **d** Pairwise alignment of the genomes of *Frankia alni* ACN14a (chromosomes) and *Frankia* sp. ACN1^ag^ (90 scaffolds) (92 % ANI). **e** Pairwise alignment of the genomes of *F. alni* ACN14a (chromosome) and *Frankia* sp. QA3 (chromosome) (91 % ANI)

Analysis of ANI showed that while *Candidatus* Frankia datiscae Dg1 and *Frankia* sp. BMG5.1 belong to the same species (96 %; see also [6]), Dg2 belongs to a different species (ca. 88 % ANI vs. Dg1/BMG5.1). To better understand the evolution of Cluster II strains, genome alignments were performed for Dg1/Dg2 (Fig. 2a) and Dg1/BMG5.1 (Fig. 2b). It should be taken into account that the genomes of Dg2 and BMG5.1 are draft genome the scaffolds of which (1066 scaffolds for Dg2, 116 for BMG5.2) were aligned with the Dg1 genome so that not all rearrangements are visible. For comparison, genome alignments were performed for two strains from the same species from Cluster Ic, i.e., the *Casuarina-*infective strains CcI3 (fully assembled chromosome; [7]) and BMG5.23 (166 scaffolds; [8]) with ANI 99 % (Fig. 2c) and for two strains from different species from *Frankia* Cluster Ia, i.e., the *Alnus*-infective strains ACN14a (fully assembled chromosome; [7]) and ACN1^ag^ (90 scaffolds; [13]) with ANI 92 % (Fig. 2d). Thus, a high amount of genome rearrangements is observed in Cluster II strains compared to the Cluster I strains, which would seem consistent with the high amount of complete IS elements in Dg1 compared to other *Frankia* strains [35]. However, a genome alignment of two *Alnus*-infective strains from different continents, ACN14a (Canada) and QA3 (Pakistan; fully assembled chromosome; [11]) with ANI 91 % shows a far higher amount of genome rearrangements than between Dg1 and Dg2 (Fig. 2e). To some extent, this can be explained by the fact that the genomes of both ACN14a and QA3 represent fully assembled chromosomes.

### Dg2 is predicted to have greater saprotrophic capabilities than Dg1     

A comparison between the ORFs found in the genomes of Dg1 and BMG5.1 versus Dg2, respectively, shows that Dg2 has the ability to use nitrate as N-source in that it can form assimilatory nitrate reductase and nitrite reductase (FDG2_0181, _0191, _0192, _0193) which are also encoded on the BMG5.1 genome but lacking in Dg1. Several transporters are present in the Dg2 metagenome that also occur in other *Frankia* strains, but are missing in Dg1 and BMG5.1, e.g., a putative ABC sugar transporter (FDG2_6040, _6041, _6042). Dg2 also contains an acetone carboxylase putatively involved in the use of acetone as carbon source which are missing in Dg1 and BMG5.1 (FDG2_0380, _0381, _0382; Clark and Ensign 1999). So Dg2 should be better equipped for saprotrophic growth than Dg1.

Furthermore, the Dg2 metagenome contains more operons for the production of secondary metabolites than either the genome of Dg1 or of BMG5.1 (Additional file 2).

### Dg2 contains the canonical *nod* genes *nodABC* linked to 2 copies of *nodH*

**Fig. 3:**
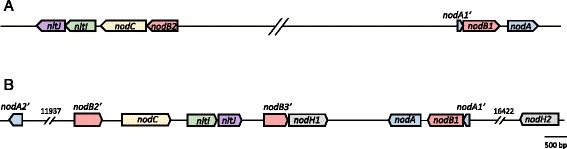
Arrangement of the canonical *nod* genes and the *nodIJ* homologs *nltIJ* in the genomes of Dg1 (**a**) *vs.* Dg2 (**b**). In Dg2, the canonical *nod* genes are clustered in a single part of the chromosome. The *nodABC* gene region of Dg2 also contains two copies of *nodH. nodA* genes are given in blue, *nodB* in red, *nodC* in yellow, *nodH* in grey, *nltI* in green and *nltJ* in purple

Like Dg1, Dg2 contains the canonical *nod* genes *nodABC*, distributed over several transcriptional units, and in a configuration very similar to that in Dg1 except that in Dg2, all *nod* genes are clustered in one area of the chromosome (Fig. 3). Sequence alignments of the encoded proteins are shown in Additional file 3. Like in Dg1, a *nodBC* operon (DG2_3264-DG2_3265; here, containing a 5’-truncated *nodB* gene) is followed by *nodIJ* homologs (*nltI* and *nltJ*, DG2_3266 and DG2_3267) and like in Dg1, the distance between the open reading frames makes it questionable whether *nodBCnltIJ* are forming an operon [35].

The presence of several truncated copies indicates a number of transposition events in the past. The configuration of the Dg2 *nodA1’nodBnodA* operon (DG2_3275 – DG2_3273) matches that in Dg1; furthermore, the 3’-truncated *nodA1’* genes at the beginning of the Dg1 and the Dg2 operon are missing the same parts and are nearly identical (Fig. 3; Additional file 3). Thus, the transpositions that led to the *nodA1’nodBnodA* configuration must have occurred in a common ancestor of Dg1 and Dg2. Dg2 contains an additional 5’-truncated copy of *nodA,* Dg2*nodA2’* (DG2_3248) which is nearly identical with Dg2*nodA1* (Fig. 3; Additional file 3) and presumably arose by incomplete duplication of Dg2*nodA1*. NodB sequence alignment shows that Dg1NodB2, encoded by the *nodB* gene of the *nodBC(nltIJ)* operon, contains an insertion in its N-terminal part while its orthologue Dg2NodB2’ (DG2_3264) is truncated in the 5’- and 3’-part (Additional file 3). Thus, a comparison of the *nod* gene region of Dg1 and Dg2 indicates that the *nodA1’nodB1nodA* operon was present in the common ancestor of both strains. In Dg1, half of the *nod* region was transferred over more than 1 Mb to another part of the chromosome. In Dg2, a partial duplication of *nodA* took place, yielding *nodA2’*, and the *nodB2* gene (part of the *nodB2C* operon) acquired a 5’- and a 3’-deletion.

In Dg2*,* the *nodABC* genes are linked to two complete copies of an additional *nod* gene, *nodH*, encoding a sulfotransferase (DG2_3270 and DG2_3293; Fig. 3). Similar to the *nodABC genes* (*nodA,* 59.10 % GC; *nodA1’,* 59.92 % GC; *nodA2’*, 60.36 % GC; *nodB1*, 66.08 % GC; *nodB2’,* 66.66 % GC; *nodB3’*, 62.76 % GC; *nodC,* 64.60 % GC; *nltI*, 60.6 %; *nltJ*, 66.5 %), both *nodH* genes have a GC content below the average of the Dg2 metagenome (*nodH1,* 60.64 % GC; *nodH2,* 61.04 % GC).

### NodH phylogeny

**Fig. 4:**
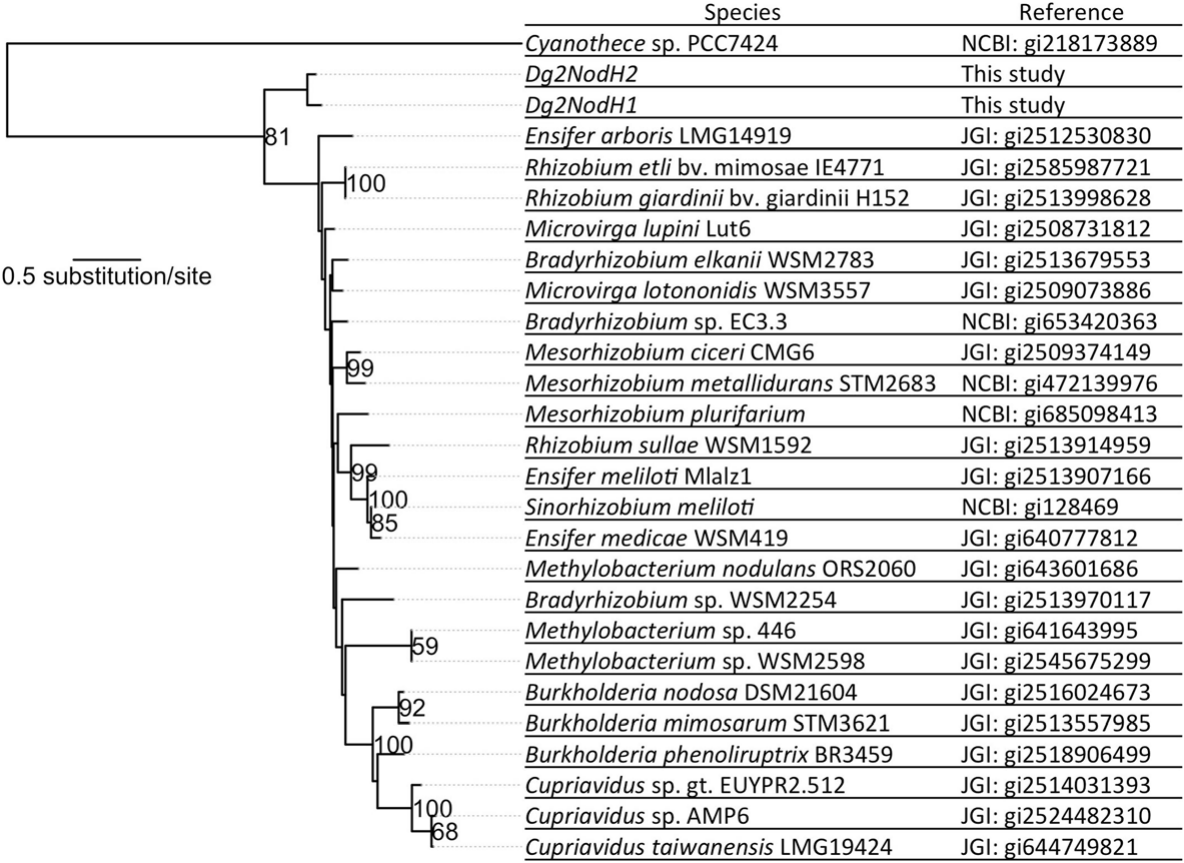
The most likely phylogeny of *nodH* genes based on amino acid sequences. Nodes with bootstrap support of 50 or higher have their values indicated at each node. The scale bar indicates the number of amino acid substitutions per site

The amino acid sequences from the two putative NodH proteins encoded by Dg2 were used in a maximum likelihood phylogenetic analysis. PROTTEST found JTT + G (α = 0.824) to be the best model based on the Bayesian information criterion (BIC) and on the corrected Akaike information criterion (AICc). GARLI2.0 generated the most likely phylogeny with a likelihood score of -6545.95. A putative sulfotransferase from *Cyanothece* sp. PCC7424 (NCBI, GI: 218173889), the only cyanobacterial sequence in the tree with the most distantly related taxa with by far the longest branch, was used to root the tree. The resulting tree (Fig. 4) shows that the two Dg2 NodH proteins form a sister group to rhizobial NodH proteins.

### *NodH* expression could not be detected in nodules

*NodH* is clearly not essential to nodulate *Coriaria nepalensis*, the original host for Dg1, or *D. glomerata*, in whose nodules Dg1 was maintained for over a decade, or *D. cannabina* which can be nodulated by Dg1 as well as by Dg2, respectively (Additional file 4). To address the question about the expression of Dg2 *nodH* in nodules*, D. glomerata* nodules induced by nodules induced by Dg2 were examined for *nodA, nodB, nodC* and *nodH* mRNA using quantitative RT-PCR. While *nodABC* were expressed, expression of *nodH* could not be detected in Dg2-induced nodules of *D. glomerata* (Additional file 5).

### Phylogeny of Cluster II *Frankia* strains – North America *vs.* Eurasia

Previously, Vandenheuvel et al. [21] had found low genetic diversity of Cluster II *Frankia* strains from western North America while using a *glnA* fragment as phylogenetic marker. Our core genome comparison shows that while Cluster II strains are not as genetically diverse as Cluster III strains, there are strong differences between Cluster II strains from Asia (represented by Dg1 and BMG5.1) and from North America (represented by Dg2; Fig. 1). Among the three Cluster II genomes available, Dg2 is sister to the two genomes from Asia, Dg1 from Pakistan and BMG5.1 from Japan [6]. In Cluster II phylogenies involving more strains, the New Zealand strains were always ancestral [32]. Therefore, without a genome from a Cluster II strain from New Zealand included, this core genome tree cannot be used as the basis to determine the earliest divergent lineage of Cluster II *Frankia.*

**Fig. 5:**
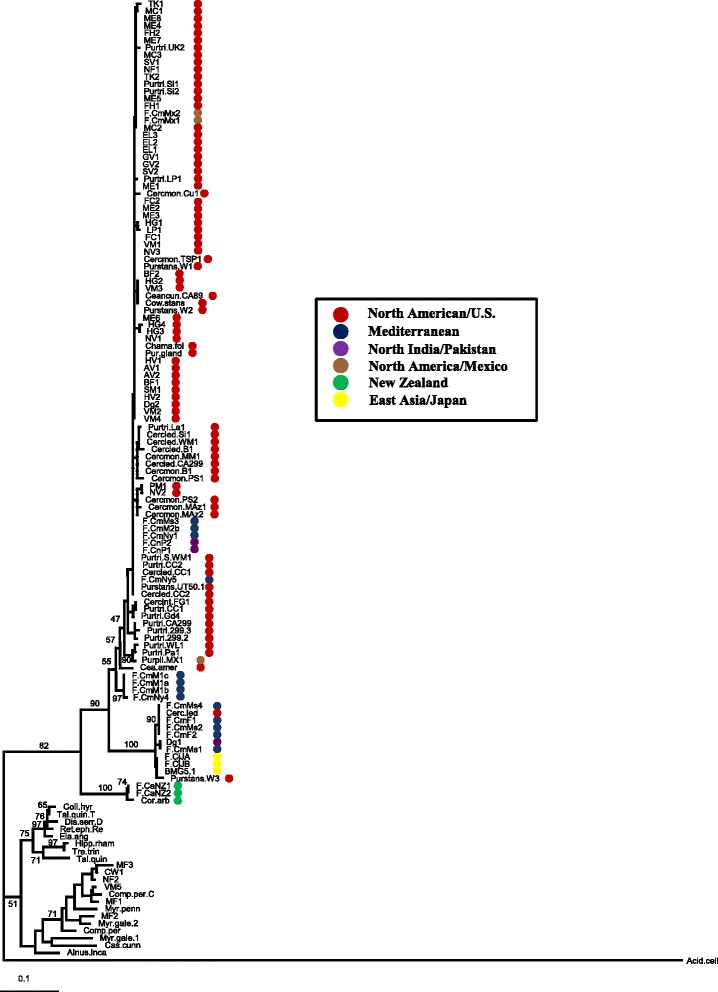
Phylogeny of Cluster II *Frankia* strains based on *glnA.* All DNA sequences used in this figure are referenced in Additional file 6. *Acidothermus cellulolyticus* [93] and several Cluster I and Cluster III strains were used as outgroups. Sequences were taken from Vanden Heuvel et al. [21], Clawson et al. [33] and Nouioui et al. [27] and from the three Cluster II genomes available; sequences of 34 field samples were contributed by this study (see Additional file 6)

When the *glnA*-based phylogeny of Vandenheuvel et al. [21] was extended to include all Cluster II strains analysed thus far (the *Coriaria*-infective strains from Nouioui et al. [27], as well as a new collection of sequences amplified from nodules of North American Dryadoideae), the diversity of the Eurasian Cluster II strains was as low as that of the North American Cluster II strains (Fig. 5; Additional file 6), a phylogenetic grouping which contradicts our core genome comparison of the two Asian strains Dg1 and BMG5.1 with Dg2 (Fig. 1). Furthermore, the *glnA*-based phylogeny did not result in an unambiguous separation of Eurasian and North American Cluster II strains. No greater diversity was found when 16S rRNA sequences were included in the comparison (data not shown). So *glnA,* while a suitable marker for the phylogeny of the whole *Frankia* genus [33], does not seem to show enough sequence diversity to reflect the phylogeny of Cluster II *Frankia* strains.

## Discussion

In this study, we sequenced the DNA from *Frankia* vesicle clusters isolated from nodules of *Datisca glomerata* inoculated using a field sample of soil from Gates Canyon, Vacaville, CA, USA. It turned out to be a metagenome (Dg2) representing two dominant (Dg2a, Dg2b) and one minor (Dg2c) *Frankia* Cluster II strains. Analysis of average nucleotide identity (ANI) revealed that Dg2 represents a new species in *Frankia* Cluster II, while the previously sequenced genomes of *Candidatus* Frankia datiscae Dg1 (originating in Pakistan; [35]) and of *Frankia* sp. BMG5.1 (originating in Japan; [6]) belong to the same species [6]. For comparison with the diversity of Cluster I *Frankia* strains, all *Casuarina*-infective strains the genomes of which were published so far belong to the same species (ANIs between 99.4 and 99.9 %), while the three *Alnus*-infective *Frankia* strains from Canada the genomes of which were sequenced represent two species: ACN14a [7] and AvcI1 [14] have 99 % ANI, while ACN1^ag^ has 92 % and 91.44 % ANI, respectively, with ACN14a and AvcI1 [14]. The *Alnus*-infective *Frankia* strain from Pakistan, QA3 [11], represents a third species, showing 91 % ANI with ACN14a, 92.97 % ANI with AvcI1 and 92 % ANI with ACN1^ag^.

*Frankia* genome comparisons have shown that members of the non-symbiotic Cluster IV and of the symbiotic Cluster III have the largest genomes with ca. 10 Mb. Genome reduction took place in the symbiotic Cluster I – strongly in *Casuarina*-infective strains where genome size is reduced to 5–6 Mb [7–10], and less strongly in *Alnus*-infective strains where genome sizes are between 7 and 8 Mb [7, 11, 13, 14]. This differential genome reduction has been correlated with the saproptrophic potential of the corresponding strains and with the phytogeographical distribution of their hosts [7], or with their host range [76, 77]. The fact that for decades, Cluster II strains could not be cultured, indicating a low saprotrophic potential, would lead to the prediction of a small genome size, while the low host specificity of Cluster II strains [21] would predict a large genome size. Since all three Cluster II genomes sequenced so far are in the range of 5–6 Mb, the saprotrophic potential seems to be the decisive factor here.

A phylogenetic tree based on core genome comparison of 16 *Frankia* strains and three actinobacterial outgroups showed that *Frankia* Cluster II is not only paraphyletic to the other symbiotic *Frankia* strains, but is also also paraphyletic to Cluster IV which contains the non-symbiotic strains (Fig. 1). While Cluster II symbioses may be the oldest root nodule symbioses [2], they cannot precede the acquisition of the predisposition for developing root nodules by the precursor of the symbiotic plant clade which is dated to ca. 100 mya.

The only cultured Cluster II strain reported to date is an alkaliphile [6]. It is highly unlikely for a root symbiont that colonizes the proton-rich rhizosphere and acidic plant endosphere, to secondarily gain alkaliphily making it intolerant of pH 6 in culture. However, this perplexing character is apparently not limited to a single strain; Canizo et al. [78] showed that while Cluster II *Frankia* nodulated *Coriaria myrtifolia* at a range of pH between 5 and 9, higher rates of nodulation and host growth correlated directly with increasing pH. Furthermore, in New Zealand, *Coriaria* sp. grows on volcanic slopes (see, e.g., [79]), and soils of active volcanic areas tend to be alkaline [80]. Considering the genome phylogeny which shows Cluster II *Frankia* as sister to all other *Frankia*, this raises the possibility that alkaliphiles predates symbiosis in *Frankia*. As the alkaliphilic and symbiotic crown group of the genus diverged into Cluster II and its sister, the sister lost its alkaliphily.

Ultimately, more Cluster II strains have to be cultured to determine whether this scenario is correct. If, however, the ancestral *Frankia* strains were alkaliphiles, Cluster II strains would not necessarily represent examples of genome reduction. Contrarily, the evolutionary more derived *Frankia* strains might represent examples of genome expansion during the adaptation to diverse moderate environments.

### How many strains are present in a typical *Frankia* Cluster II-induced nodule?

Studies on the diversity of *Frankia* in root nodules have usually relied on the PCR amplification of 16S rRNA fragments (see, e.g., [81]). Since actinorhizal nodules have thick periderms, sequences amplified that way do not necessarily derive from the microsymbionts of said nodules, but may be derived from bacteria living in the dead cells of the periderm and having escaped surface sterilization. This may also be exemplified by the fact that not all strains isolated from nodules of a particular plant can re-infect that plant species (see, e.g., [82]).

The genomes of two strains have now been sequenced based on DNA derived from vesicle cluster preparations from nodules of several plants. Earlier results based on the analysis of OTUs from nodules had shown that greenhouse-grown nodules of *Datisca glomerata* contained more than one *Frankia* strain, although only one strain, *Candidatus* Frankia datiscae Dg1, was represented in vesicle clusters [35]. Our study shows the first *Frankia* metagenome isolated from *D. glomerata* nodules induced by a field sample from *D. glomerata,* Dg2. The difference with Dg1 may have been due to the fact that Dg1 originated from soil below a nodulated *Coriaria nepalensis* plant and was propagated for more than ten years in *D. glomerata* plants in a greenhouse [35], while Dg2 is based on soil collected from below a nodulated *D. glomerata* plant in the field that was used to nodulate a single series of *D. glomerata* plants in a growth chamber before the genome was analysed. Thus, Dg2 is likely to better represent the situation in the field. Nevertheless, further studies on vesicle clusters isolated from *Frankia* Cluster II nodules in particular, and *Frankia* nodules in general, are necessary to find out how many strains are normally represented as symbionts in the nodules of an individual actinorhizal plant. Another question to be analysed in the future is whether the wide host range of *Frankia* Cluster II is due to the fact that different members of an assemblage dominate in nodules from different host species.

### Dg2 contains the canonical *nod* genes and also the sulfotransferase gene *nodH:* does it produce sulfated Nod factors?

Bacterial signaling in rhizobial symbioses as well as fungal signaling in arbuscular mycorrhizal symbioses [39], and also *Frankia* signaling in actinorhizal symbioses [40, 41, 83] involves the common symbiotic signal transduction pathway (CSSP). This pathway has been well examined in legumes, where rhizobial or fungal lipochitooligosaccharide (LCO) signal factors bind to modified chitin receptors in the plasma membrane of root epidermal cells which signal to a common symbiotic receptor kinase SymRK/DMI2 [38, 39]. The modified chitin receptors belong to a protein family that meanwhile has been shown to perceive rhizobial LCO Nod factors, LCO Myc factors from arbuscular mycorrhizal fungi, peptidoglycans and exopolysaccharides [84]. Studies on *Alnus glutinosa* and *Casuarina glauca* have shown that in symbioses of *Frankia* Cluster I, non-LCO signal substances are signaling via the CSSP [83, 85, 86]. This is consistent with the fact that Cluster I (and Cluster III) *Frankia* strains do not contain the canonical *nod* genes *nodABC* which encode the enzymes that in rhizobia are responsible for the biosynthesis of LCO Nod factors [7]. Similarly, some rhizobial strains that do not contain the canonical *nod* genes and cannot form LCO Nod factors can induce nodules on *Aeschynomene* sp., and also this process involves signaling via the CSSP [87].

So far, one *Frankia* genome was known to contain the canonical *nod* genes *nodABC*, Dg1 [35]. Dg2a and Dg2b not only contained the canonical *nod* genes but also two copies of the *nodH* gene encoding a sulfotransferase which in rhizobia is responsible for the production of sulfated LCO Nod factors. As in case of Dg1, the canonical *nod* genes of Dg2 could be shown to be expressed in nodules, but neither *nodH* copy was transcribed under these conditions. A comparative analysis of the *nod* operons showed that the common ancestor of Dg1 and Dg2 – which should also be the ancestor of BMG5.1 – is likely to have contained the canonical *nod* genes *nodABC* and probably also a copy of *nodH* (Fig. 3; Additional file 3).

While the presence of a diverse NodA protein family in actinobacteria suggested that the corresponding gene evolved in actinobacteria and was later picked up by rhizobia, maybe as an operon together with *nodB* and *nodC* [35], the situation is not clear for *nodH*. In the absence of a NodH protein family in non-symbiotic relatives of either *Frankia* sp. or rhizobia, the fact that phylogenetically, Dg2 NodH proteins are forming a sister group of rhizobial NodH proteins (Fig. 4) merely indicates either that a gene transfer took place between rhizobia and *Frankia* strains, or that both groups obtained *nodH* genes from the same donor.

The high number of full size insertion elements in Dg1 [35] and the clear signs of transposition in the *nod* gene regions of Dg1 and Dg2, respectively, indicate significant genome instability. Under these circumstances, the fact that both Dg1 and Dg2 contain an intact copy of *nodA, nodB* and *nodC* each seems to indicate selection pressure, and thus a physiological function of *nodABC.* This does not necessarily lead to the conclusion that nodulation by Dg1 and Dg2, or by one of them, requires LCO Nod factors. Nevertheless, the fact that *nodABC* are expressed in nodules induced by Dg1 and Dg2 suggests that LCOs do play a role in the symbiosis.

Sulfation of LCO Nod factors, apart from playing a role in host specificity, can increase the stability of Nod factors by interfering with their digestion by chitinases [88]. This is important in the rhizosphere but may not be relevant for Nod factors *in planta*. So if sulfation of Nod factors does not play a role in binding Nod factor receptors within *D. glomerata* nodules, there might be a rationale for not expressing *nodH* genes *in planta,* only *ex planta.* It is also possible that the *nodH* genes are not expressed in *D. glomerata* nodules because they are not necessary to nodulate this particular host species, as exemplified by the fact that *D. glomerata* is nodulated by Dg1 which does not contain *nodH.*

### Other unique features of *Frankia* Cluster II strains: mammalian cell entry genes

With three genomes of Cluster II available, it becomes interesting to do phylogenetic profiling to identify genes that only occur in Cluster II, not in any other *Frankia* cluster. Most strikingly, what is exclusive to Cluster II genomes are large sets of *mce* (mammalian cell entry) genes. They represent the actinobacterial paralogues of the *Mla* systems (ABC-type transporters; *mlaD* encodes the periplasmic substrate-binding proteins, *mlaE* encodes the permease and *mlaF* the ATP-binding protein) of Gram-negative bacteria that maintain asymmetry in the outer membrane [89]. *Mce* genes were first analysed in *Mycobacterium tuberculosis. Mce* systems seem to represent steroid transporters in that *Mycobacterium* spp*. Mce4* facilitates the uptake of cholesterol and also in *Rhodococcus jostii* RHA1, *mce4* encodes an ATP-dependent steroid transporter necessary for growth on, e.g., beta-sitosterol [90]. They are involved in virulence as could be shown since peptides that strongly bind, and thereby block, the periplasmic component of the *mce4* system from *M. tuberculosis* could prevent the mycobacterial entry to type II pneumocytes [91]. In *Streptomyces coelicolor,* the *mce* locus is involved in mediating the interactions with plants and amoebae. *Mce* genes are present across the actinobacteria and presumably represent an ancient locus; niche specialization has led to divergence of the *mce* clusters in each actinobacterial genus throughout evolution [92].

Analysis showed that the *mce* systems in the three representatives of *Frankia* Cluster II are highly diverse (Additional file 7). Several *Frankia* Cluster II *mce* operons contain truncated genes. One operon is present in BMG5.1 and Dg2, but not in Dg1, although it is conserved in other actinobacteria, i.e. in *Saccharomonospora marina*, suggesting that Dg1 lost the entire operon. In Dg2, this operon mostly contains truncated genes (FDG2_3167 – FDG2_3173; Additional file 7). Furthermore, Dg1 does not contain a *mlaF* homolog but this component might be provided by another ABC transporter system. Every Cluster II strain contains at least two *mlaE* genes (encoding permeases) and more than 10 *mlaD* genes (encoding the periplasmic substrate-binding proteins). Given that the *mce* systems are involved in the colonization of plants in case of *S. coelicolor,* it is tempting to speculate that they might be involved in the infection process of *Frankia* Cluster II strains.

### How did *Frankia* Cluster II invade the North American continent?

A *glnA*-based phylogenetic tree of all Cluster II *Frankia* sequences available to date with Cluster I and III sequences and the *glnA* sequence from *Acidothermus cellulolyticus* [93] as outgroup (Fig. 5) had the three sequences from New Zealand in the ancestral position but the Eurasian and North American strains did not form separate clades in that support for the separation was weak and several strains ended up in the ‘wrong’ group (American strains in the Eurasian group and *vice versa*). However, if the Cluster II strains nodulating the North American host plants (Dryadoideae, *Ceanothus* sp. and *D. glomerata*) were derived from South American strains which originally came from New Zealand [16], we would not expect the separation between the New Zealand strains and the Northern Hemisphere strains we see in Fig. 5. So the currently available are more consistent with the hypothesis that *Frankia* Cluster II strains reached the North American continent with *D. glomerata via* the Bering strait.

## Conclusions

The first metagenome of an assemblage of North American *Frankia* Cluster II strains, Dg2, represents a different species than the two Asian strains. A phylogenetic tree based on the core genomes of 16 *Frankia* strains puts Cluster II in the basal position of the entire genus, and shows that the Cluster II genomes available thus far display more diversity than the *Casuarina*-infective strains from Cluster I. The Dg2 metagenome contains not only the canonical *nod* genes *nodABC,* but also the LCO sulfotransferase gene *nodH*, and a comparison between the *nod* regions of *Candidatus* Frankia datiscae Dg1 from Pakistan and Dg2 from California shows that both strains have a common ancestor the genome of which probably contained *nodH*.

## Additional files

Additional file 1: Contig-length vs. Read-count plot of the Dg2 metagenome. (DOCX 348 kb)
Additional file 2: Secondary metabolite production encoded on genomes of F*rankia* Cluster II strains. (XLSX 12 kb)
Additional file 3: Alignments of Nod DNA and protein sequences from Cluster II *Frankia* strains Dg1 and Dg2. (DOCX 42 kb)
Additional file 4:
*Candidatus* Frankia datiscae Dg2-induced nodules on roots of *D. glomerata* and *D. cannabina*. (PPTX 5298 kb)
Additional file 5: Expression of *nod* genes in nodules induced by Dg2 on roots of *D. glomerata*. (DOCX 13 kb)
Additional file 6:
*GlnA* sequences used for the phylogenetic tree depicted in Fig. 5. (XLSX 26 kb)
Additional file 7: MCE-related proteins encoded by the genomes of BMG5.1, Dg1 and Dg2. (XLSX 26 kb)

## Abbreviations

ANI: Average nucleotide identity
CSSP: Common symbiotic signal transduction pathway
LCA: Lowest common ancestor (algorithm)
LCO: Lipochitooligosaccharide
Mya: million years ago
Nod factors: Nodulation factors
PCR: Polymerase chain reaction
RT-qPCR: Reverse transcription quantitative real-time PCR
SNP: Single nucleotide polymorphism
