# Supplementary material for: An assemblage of Frankia Cluster II strains from California contains the canonical nod genes and also the sulfotransferase gene nodH
Source: BMC Genomics. 2016 Oct 12;17:796. doi: 10.1186/s12864-016-3140-1 (PMC5059922; doi:10.1186/s12864-016-3140-1)
Supplement: Additional file 1: — Contig-length vs. Read-count plot of the Dg2 metagenome. (DOCX 348 kb) [file 12864_2016_3140_MOESM1_ESM.docx]

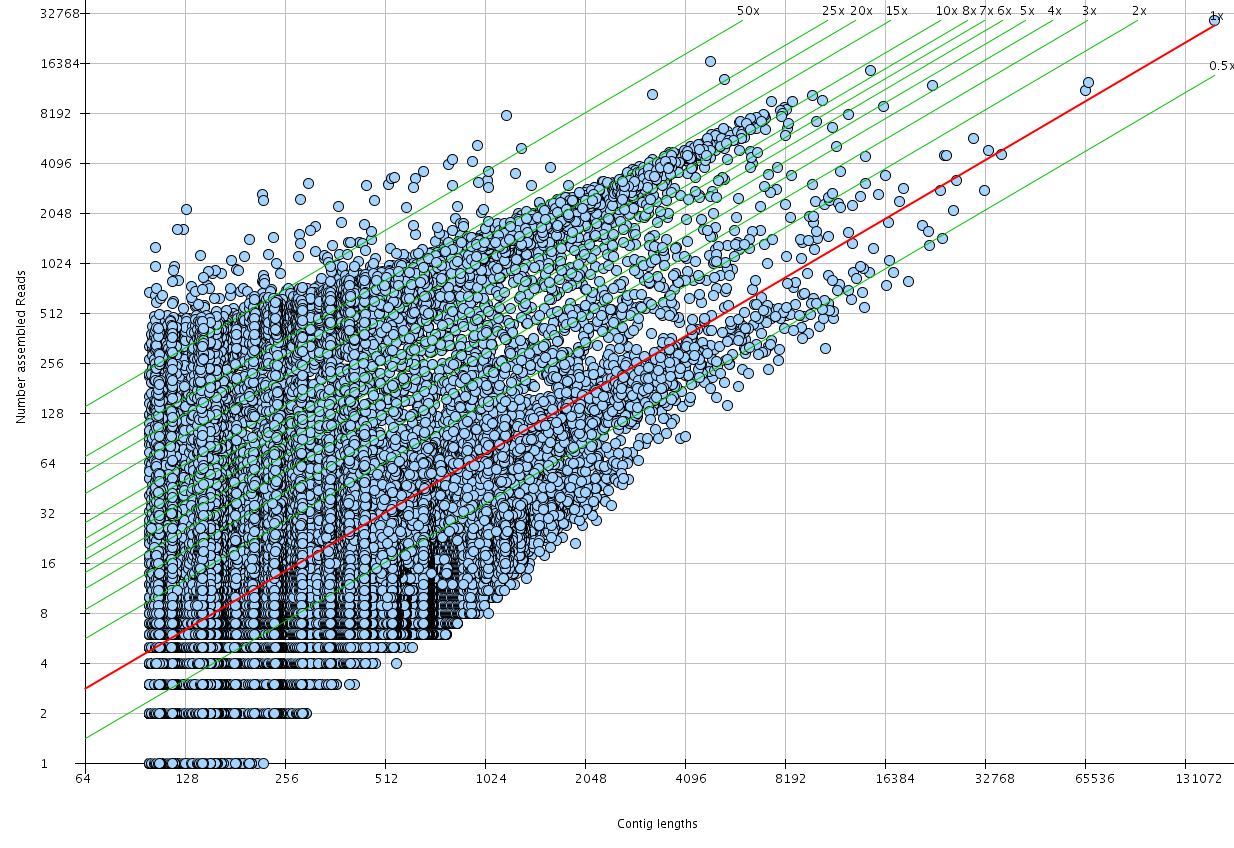


**Additional file 1. Contig-length vs. Read-count plot of the Dg2 metagenome.** The length of contigs (x-axis) was plotted as a function of the number of reads assembled into each contig (y-axis). Lines indicate the predicted coverage of contigs within the genome. The red line represents the calculated coverage of one-fold contigs. The different points represent different contigs. Two lines of points can be seen in the plot around the red line. The more abundant line of points represents the two abundant *Frankia* strains Dg2a and Dg2b, whereas the low abundant line represents the third *Frankia* strain Dg2c, which is underrepresented in the sample.
