# Supplementary material for: An assemblage of Frankia Cluster II strains from California contains the canonical nod genes and also the sulfotransferase gene nodH
Source: BMC Genomics. 2016 Oct 12;17:796. doi: 10.1186/s12864-016-3140-1 (PMC5059922; doi:10.1186/s12864-016-3140-1)
Supplement: Additional file 3: — Alignments of Nod DNA and protein sequences from Cluster II Frankia strains Dg1 and Dg2. (DOCX 42 kb) [file 12864_2016_3140_MOESM3_ESM.docx]

**A**

* * * * * *
**Dg1*nodA***  1 ATGAAAACGCAAATTCAGTGGAAAACGTACTGGGAGAATCAGCTCGAACCGGGTGACCAC 60
**Dg2*nodA***  1 ATGAAAACGC**A**AATTCAGTGGAAAACGTACTGGGAGAATCAGCTCGAACCGGGTGACCAC 60
**Dg2*nodA1’*** - ------------------------------------------------------------ -
**Dg2*nodA2’*** 1 ATGAATACCGAAATCCAGTGGCGGATATGCTGGGAGGCCCAGCTTACGCCGACTGATCAC 60
**Dg1*nodA’*** 1 ATGAATACCGAAATCCAGTGGCGGATATGCTGGGAGGACCAGCTTACGCCGACTGATCAC 60

 * * * * * *
**Dg1*nodA***  61 GCGGAACTTGCCGAGTTTTTTCGAAACACCTACGGAGCTGTGGGTGCCTGGAACGTGAAA 120
**Dg2*nodA*** 61 GCGGAACTTGCCGAGTTTTTTCGAAACACCTACGGAGCTGTGGGTGCCTGGAACGTGAAA 120
**Dg2*nodA1’*** - ------------------------------------------------------------ -
**Dg2*nodA2’***  61 ------------------------------------------------------------ 61
**Dg1*nodA’*** 61 ------------------------------------------------------------ 61

 * * * * * *
**Dg1*nodA*** 121 CATTTCAAAGGTAGCCGTAGCTGGGCCGGAGCGCGACCCGAGCTCAGGATAATCGGGCAT 180
**Dg2*nodA*** 121 CATTTCAAAGGTAGCCGTAGCTGGGCCGGAGCGCGACCCGAGCTCAGGATAATCGGGCAT 180
**Dg2*nodA1’***  - ------------------------------------------------------------ -
**Dg2*nodA*2*’***  61 ------------------------------------------------------------ 61
**Dg1*nodA’*** 61 ------------------------------------------------------------ 61

 * * * * * *
**Dg1*nodA*** 181 GACGAGCGCGGAGTGGCGGCGCACTTCGGGGTGCTGCGTCGTTTTATCAGGCTCGGCGCA 240
**Dg2*nodA*** 181 GACGAGCGCGGAGTGGCGGCGCACTTCGGGGTGCTGCGTCGTTTTATCAGGCTCGGCGCA 240
**Dg2*nodA1’*** - ------------------------------------------------------------ -
**Dg2*nodA2’*** 61 ------------------------------------------------------------ 61
**Dg1*nodA’*** 61 ------------------------------------------------------------ 61

 * * * * * *
**Dg1*nodA*** 241 ATCGATCAGCTCGTCGCCGAGGTCGGGCTGTACGCTGTACGCCCGGATCTACAGCGATCG 300
**Dg2*nodA***  241 ATCGATCAGCTCGTCGCCGAGGTCGGGCTG**T**ACGCTGTACGCCCGGATCTACAGCGATCG 300
**Dg2*nodA1’*** - ------------------------------------------------------------ -
**Dg2*nod*A2*’*** 61 ------------------------------------------------------------ 61
**Dg1*nodA’*** 61 ------------------------------------------------------------ 61

 * * * * * *
**Dg2*nodA*** 301 GGAATCGGTTATCAATCCGGGATCAACGTGATGATTCCGACGCTGCTGGACCTGAAAGTC 360
**Dg1*nodA*** 301 GGAATCGGTTATCAATCCGGGATCAACGTGATGATTCCGACGCTGCTGGACTTGAAGGTC 360
**Dg2*nodA1’***  1 ------------------------------ATGATTCCGACGCTGCTGGACCTGAAAGTC 30
**Dg2*nodA2’*** 61 ------------------------------------------------------------ 61
**Dg1*nodA’*** 61 ------------------------------------------------------------ 61

 * * * * * *
**Dg1*nodA*** 361 CCGTTCGGTTTCGGAACCGTTCGCCACGAGTTGCGCGAGCATGTCGAGAAGGCCAGCGCT 420
**Dg2*nodA***  361 CCGTTCGGTTTCGGAACCGTCCGCCACGAGTTGC**G**CGAGCATGTCGAGAAGGCCAGCGCT 420
**Dg2*nodA1’*** 31 CCGTTCGGTTTCGGAACCGTCCGCCACGAGTTGCGCGAGCATGTCGAGAAGGCCAGCGCT 90
**Dg2*nodA2’*** 61 ------------------------------------------GTGGAGCCGGCG------ 72
**Dg1*nodA’***  61 ------------------------------------------GTGGAGTCGGCG------ 72

 * * * * * *
**Dg1*nodA*** 421 GCGCTGATGATCGTGTCCGGAGTCCTGGTAAGGTCTACAAATCTGTGGGACAAGTCCAAG 480
**Dg2*nodA***  421 GCGCTGATGATCGTGTCCGGAGTCCGGGTAAGGTCTACAGATCTGTGGGACAAGTCCAAG 480
**Dg2*nodA1’***  91 GCGTTGATGATCGTGTCCGGGGTCCTGGTGAGGTCTACAAATCTGTGGGACAAGTCCAAG 150
**Dg2*nodA2’*** 73 ------------------------------------------------GAACGGGAGACG 84

**Dg1*nodA’***  73 ------------------------------------------------GAACGGGAGACG 84

 * * * * * *
**Dg1*nodA***  481 ACGTGTATCGAAGATCTTCTCGTCCTGGTCATTCCGCTTGAGCGTGCCGTGGACGAGTGG 540
**Dg2*nodA***  481 ACGTGTGTCGAAGATCTT**C**TCGTCCTAGTCATTCCGCTTGAGCGCGCCGTGGACGAGTGG 540
**Dg2*nodA1’*** 151 ACGTGTCTCGAAGATCTTCTCGTCCTAGTCATTCCGCTTGAGCGCGCCGTGGACGAGTGG 210
**Dg2*nodA2’*** 85 GTGTGTC----------------------------------------------------- 91
**Dg1*nodA’*** 85 GTATGTC----------------------------------------------------- 91

* * * *
**Dg2*nodA*** 541 CCGGCCGGAGAGCTCATCGACCGGAACGGACCCGAGCTGTGA 582
**Dg1*nodA*** 541 CCGGCCGGAGAGCTCATCGACCGGAACGGACCCGAGCTGTGA 582
**Dg2*nodA1’*** 211 CCAGCCGGAGAGCTCATCGACCGGAACGGACCCGAGCTGTGA 252
**Dg2*nodA2’*** 92 ----------------------GGAAGGAGCCCGAACTGTGA 111
**Dg1*nodA’***  92 ----------------------GGAAGGAACCCGAACTGTGA 111

* * * * * *
**Dg1NodA**  1 MKTQIQWKTYWENQLEPGDHAELAEFFRNTYGAVGAWNVKHFKGSRSWAGARPELRIIGH 60

**Dg2NodA**  1 MKTQIQWKTYWENQLEPGDHAELAEFFRNTYGAVGAWNVKHFKGSRSWAGARPELRIIGH 60
**Dg2NodA1'** - ------------------------------------------------------------ -
**Dg2NodA2'** 1 MNTEIQWRICWEAQLTPTD----------------------------------------- 19
**Dg1NodA'**  1 MNTEIQWRICWEDQLTPTD----------------------------------------- 19

 * * * * * *
**Dg1NodA**  61 DERGVAAHFGVLRRFIRLGAIDQLVAEVGLYAVRPDLQRSGIGYQSGINVMIPTLLDLKV 120

**Dg2NodA**  61 DERGVAAHFGVLRRFIRLGAIDQLVAEVGLYAVRPDLQRSGIGYQSGINVMIPTLLDLKV 120

**Dg2NodA1'** 1 --------------------------------------------------MIPTLLDLKV 10
**Dg2NodA2'** 61 ------------------------------------------------------------ 61
**Dg1NodA'** 61 ------------------------------------------------------------ 61


 * * * * * *
**Dg1NodA**  121 PFGFGTVRHELREHVEKASAALMIVSGVLVRSTNLWDKSKTCIEDLLVLVIPLERAVDEW 180
**Dg2NodA**  121 PFGFGTVRHELREHVEKASAALMIVSGVRVRSTDLWDKSKTCVEDLLVLVIPLERAVDEW 180

**Dg2NodA1'**  11 PFGFGTVRHELREHVEKASAALMIVSGVLVRSTNLWDKSKTCLEDLLVLVIPLERAVDEW 70

**Dg2NodA2'** 20 -------------HVEPA------------------ERETVC------------------ 30

**Dg1NodA'** 20 -------------HVESA------------------ERETVC------------------ 30

*
**Dg2NodA**  181 PAGELIDRNGPEL 193
**Dg1NodA**  181 PAGELIDRNGPEL 193

**Dg2NodA1'**  71 PAGELIDRNGPEL 83
**Dg2NodA2'** 31 -------RKEPEL 36
**Dg1NodA'** 31 -------RKEPEL 36

**B**

* * * * * *
**Dg1*nodB1*** 1 ---GTGACCGGATGTTGCCCGTCCAGCACGTTTTCCGGTGGTCAGT-------------- 43
**Dg2*nodB1*** 1 ---GTGACCGGACGTTGCCCGGCCAGCACGTTTTCCGGTGGTCAGT-------------- 43
**Dg1*nodB2*** 1 TTGCCGGACGGACAGACCGTGGCATCGACCACCACGGGAACGACCACGTGCCCGCAGGCC 60
**Dg2*nodB2***' - ------------------------------------------------------------ -
**Dg2*nodB3***' 1 ---GTGACACGCGCCCACCCGGCTCCAAATACGGTCGGCGACGAGC-------------- 43

 * * * * * *
**Dg1*nodB1*** 43 ------------------------------------------------------------ 43
**Dg2*nodB1*** 43 ------------------------------------------------------------ 43
**Dg1*nodB2*** 61 TACGACGACCGGCGGACCGAGGCACGCCGCACTGCAGCGGCCCTGCGCGCGCACGCCGGG 120
**Dg2*nodB2***' - ------------------------------------------------------------ -
**Dg2*nodB3***' 43 ------------------------------------------------------------ 43

 * * * * * *
**Dg1*nodB1*** 43 ------------------------------------------------------------ 43
**Dg2*nodB1*** 43 ------------------------------------------------------------ 43
**Dg1*nodB2*** 121 ACACTCAT**C**GCCACCGCGACGATGACGATCCGCTCCCGGGCCCCGGTCAGCATCTCCACC 180
**Dg2*nodB2***' - ------------------------------------------------------------ -
**Dg2*nodB3***' 43 ------------------------------------------------------------ 43

 * * * * * *
**Dg1*nodB1*** 44 ----------------------------------------------------CAGGGACT 51
**Dg2*nodB1*** 44 ---------------------------------CAGGGACTCGCCGCATCGCCAGGGACT 70
**Dg1*nodB2*** 181 CCGCTGCGGCTGGAGTCCGAACCCGGCCAGATCGACAGCCGCAAGATCCGAATCGA**A**GAG 240
**Dg2*nodB2***' - ------------------------------------------------------------ -
**Dg2*nodB3***' 44 ----------------------------------------------------CTGGAGCC 51

 * * * * * *
**Dg1*nodB1*** 52 CGCCGCATCGCCC------TCACGTTCGACGACGGACCGGATCCTTATTACACCCCCCGG 105
**Dg2*nodB1*** 71 CGCCGCATCGCCC------TCACGTTCGACGACGGACCGGATCCTTATTACACCCCCCGG 124
**Dg1*nodB2*** 241 GGCAGCCGAGACATTTTTTTGACGTTCGATGACGAACCGAACCCTTTTTGCACACCGCAG 300

**Dg2*nodB2***' - ------------------------------------------------------------ -
**Dg2*nodB3***' 52 CGCCGCATCGTCCTCGCGTTCGCGTTCGACGACGGACCAGACCCAGGCTATACACTCCTG 111

 * * * * * *
**Dg1*nodB1*** 106 ATTCTCGATTTGCTCGCTGAGCATAAGGTGGCCGCGACATTCTGCGTGCTCGGCACCTAC 165
**Dg2*nodB1*** 125 ATTCTCGATCTGCTCGCTGAGCATGAGGTGGCCGCGACATTCTGCGTGCTCGGCACCTAC 184
**Dg1*nodB2*** 301 GTACTCGATGTGCTCGCGGAGCACCGGGTAGTCGCCACCTTCTGCGTGATCGGGGAGTAT 360
**Dg2*nodB2***' 1 ---------GTGCTCGCGGAGCGCCGGGTGGCCGCCACCTTCTGCGTGATCGGGGAGCAT 51
**Dg2*nodB3***' 112 ATCCTCGATCTATTCGCTGAGCACGAGGTCCTCGCAATCTCCTGCGTGGTCGGCAGGCAT 171

 * * * * * *
**Dg1*nodB1*** 166 GCAGCCGCACATCCCGATATCATCTCCAGGATAGCCGCGGAGAGCCATCTCCTGGCCTGC 225
**Dg2*nodB1*** 185 GCAGCCGCACATCCCGATATCATCTCCAGGATAGCCGTGGAGGGCCATCTCCTGGCCTGC 244
**Dg1*nodB2*** 361 GCGG**C**CAAGCATCC**C**GAATTGATCCGGAGAATCGCCACGGAAGGCCATGGCCTGGCCAAC 420
**Dg2*nodB2***' 52 GCGGCCAAGCATCCCGAATTGATCCGGAGAATCGCCGCGGAAGGCCATGGCCTGGCCAAC 111
**Dg2*nodB3***' 172 GCAGCCGCGCATCTCGAAATTATCTCCCGGATGGTCGCGAGCGGGCATCTGCTAGCTGGC 231

 * * * * * *
**Dg1*nodB1*** 226 CACTCGATGACACACGCCGATCTGTCGTGCTGTGG--CGAGCAACAGACACGGTGGGAAA 283
**Dg2*nodB1*** 245 CACTCGATGAC**A**CACGCCGATCTGTCGCGCTGTGG--CGAGCAACAGACACGGTGGGAAA 302
**Dg1*nodB2*** 421 **C**ACACCATGACCCACCGAGACCTGTCCCGATGCGAGCCGGGCGA--GGTTCGACGGGAGA 478
**Dg2*nodB2***' 112 CACACCATGACCCACCGAGACCTGTCCCGGTGCGAGCCGGGCGA--GGTTCGACGGGAGA 169
**Dg2*nodB3***' 232 CACTCGATGACACACAGGAGCCTGTCGCGCTGTGA--CGAACAACGGACACGATGGGAAA 289

 * * * * * *
**Dg1*nodB1*** 284 TAATGGCGGCGAGCCGGACACTGCAGGCTGTCGCTCCCACCGCGCGGGTGCAGTATCTAC 343
**Dg2*nodB1*** 303 TAATGGCGGCGAGCCGGACACTGCAGGCTGTCGCTCCCACCGCGCGGGTGCAGTATCTAC 362
**Dg1*nodB2*** 479 TATCCGACGCGAACAAAGTCATCAGGACCGTTTGCCCGCAGGCCTGCGTGCATTACCTGC 538
**Dg2*nodB2***' 170 TATCCGACGCAAACACAATCATCAGGACCGTTTGCCCGCAGGCCTGCGTGCATTACTTAC 229
**Dg2*nodB3***' 290 TAAGGGAGGCGAGGCGCGCACCCTTAACGGCCACC-----------AGGGTCTTGTCTCC 338

 * * * * * *
**Dg1*nodB1*** 344 GCACACCGTATGGCCGGTGGAACGACGG-GGCGCGGCGCGTGGCGGCCGAACTCGGCCTG 402
**Dg2*nodB1*** 363 GCAC**A**CCATATGGCCGGTGGAACGACGG-GGCGCGGCGCGTGGCGGCCGAACTCGGCCTG 421
**Dg1*nodB2*** 539 GAGCGCCGTACGGCGCCTGGACCGGC**GA**-GGCCCGG---GCGGCGGCGCTGTTCGGACTG 594
**Dg2*nodB2***' 230 AGACGCCGTACAGCGCCTGGACCAGCGA-GGCCCGG---GCGGCGGCGCTGTTCGGACTG 285
**Dg2*nodB3***' 339 GGTC----TTCCGCTGCTGGA--GGCGGTGGCCCGGCCGCCGGCACACCGAT--GGGATA 390

 * * * * * *
**Dg1*nodB1*** 403 CAGCCTCTCG----GCTGGACGATAGATTCGC-GTGACTGGTCGGCACCAGGGGTGACAG 457
**Dg2*nodB1*** 422 CAGCCTCTCG----GCTGGACGATAG**ACT**CGC-GTGACTGGTCGGCACCGGGGGTGACAG 476
**Dg1*nodB2*** 595 GAGCCCCTGA----ACTGGTCGGTGGATCCGC-GCGACTGGTCACGTCCCGGCGCGAATG 649
**Dg2*nodB2***' 286 GAGCCCCTGA----ACTGGTCAGTA-ATCCGC-GCGACTGGTCGCGTCCCGGCGTGA--- 336
**Dg2*nodB3***' 391 CAGCTGGTAAGGGCACCGGCCTGCGAAGCCGCGGCGACTGAC------------------ 432

 * * * * * *
**Dg1*nodB1*** 458 AAATCATGGACGCCCTCCGGCAGCAGCTCCATCCGGGTGGGATCGTACTGCTACATGACG 517
**Dg2*nodB1*** 477 AAATCATGGACGCCCTCCGGCAACAGCTCCATCC**G**GGTGGGATCGTGCTGCTACATGACG 536
**Dg1*nodB2*** 650 TGATCGTCGAGACGGTGCTGACGTGCATCCGGCCGGGCGGAGTCATTCTTCTGCGTGGCG 709
**Dg2*nodB2***' 337 ------------------------------------------------------------ 337
**Dg2*nodB3***' 433 ----------------------------------------AACCAAACCGCCGCTCGCAC 452

* * * * * *
**Dg1*nodB1*** 518 GATGCCTGCCCGAAAGGAAAC---GTGGCTGCAGCGCCGGTCACCAAGACCAGACACTGG 574
**Dg2*nodB1*** 537 GATGTCTGCCCGAAAGGAAAC---GTGGCTGCAGCGCCGGTCACCAAGACCAGACGCTGG 593
**Dg1*nodB2*** 710 GGTGTCCGGCGGATGAGTGGCCGAGTGGCAGCCGCACCGGGCTGCGTGAACGGACAGTCA 769
**Dg2*nodB2***' 336 ------------------------------------------------------------ 336
**Dg2*nodB3***' 453 GAGATCCGATAGGTCAGAATCAATG----------------------------------- 477

 * * * * * *
**Dg1*nodB1*** 575 CTGTGGTGCCGCTTCTCATCCGCGAGCTACGCCACCAGGGCCTCGTCCCCGGTCCTCCGC 634
**Dg2*nodB1*** 594 CTGTGGTGCCGCTTCTCATCCGCGAGCTACGCCACCAGGGCCTCGTCCCCGGTCCTCCGC 653
**Dg1*nodB2*** 770 TAGCGCTCCGCCGGCTGATTCCGGCGCTGCATGAGTACGGGTTTGTCTTCCAGCCGCTTC 829
**Dg2*nodB2***' 336 ------------------------------------------------------------ 336
**Dg2*nodB3***' 477 ------------------------------------------------------------ 477

 * * * * *
**Dg1*nodB1*** 635 TGCTGGAGGCGCTGCCCCGGCTGCCGGCGGCCGGCACACCGATGGGGTACGGCTGGTAA 693
**Dg2*nodB1*** 654 TGCTGGAGGCGCTGCCCCGGCTGCCGGCGGCCGGCACACCGATGGGGTACGGCTGGTAA 712
**Dg1*nodB2*** 830 -----------CCGCCCCGTCCGTCCATTCGCCGATCAGCAGGCCGGTCTGA------- 870
**Dg2*nodB2***' 336 ----------------------------------------------------------- 336
**Dg2*nodB3***' 478 ----------------CCAGCCACAGACGAGTTGCTGAATGAAATGGAATGA------- 513

* * * * * *
**Dg1*NodB1*** 1 -MTGCCPSSTFSGG---------------------------------------------- 13
**Dg2*NodB1*** 1 -MTGRCPASTFSGG---------------------------------------------- 13
**Dg1*NodB2*** 1 MPDGQTVASTTTGTTTCPQAYDDRRTEARRTAAALRAHAGTLIATATMTIRSRAPVSIST 60
**Dg2*NodB2***' - ------------------------------------------------------------ -
**Dg2*NodB3***’ 1 -MTRAHPAPNTVGD---------------------------------------------- 13

 * * * * * *
**Dg1*NodB1*** 14 ------------------QSGTRRIAL--TFDDGPDPYYTPRILDLLAEHKVAATFCVLG 53
**Dg2*NodB1*** 14 ------------------QSGTRRIAL--TFDDGPDPYYTPRILDLLAEHEVAATFCVLG 53
**Dg1*NodB2*** 61 PLRLESEPGQIDSRKIRIEEGSRDIFL--TFDDEPNPFCTPQVLDVLAEHRVVATFCVIG 118
**Dg2*NodB2***' - ---------------------------------------------MLAERRVAATFCVIG 15
**Dg2*NodB3***’ 14 ------------------EPGARRIVLAFAFDDGPDPGYTLLILDLFAEHEVLAISCVVG 55

 * * * * * *
**Dg1*NodB1*** 54 TYAAAHPDIISRIAAESHLLACHSMTHADLSCCGEQQTRWEIMAASRTLQAVAPTARVQY 113
**Dg2*NodB1*** 54 TYAAAHPDIISRIAVEGHLLACHSMTHADLSRCGEQQTRWEIMAASRTLQAVAPTARVQY 113
**Dg1*NodB2*** 119 EYAAKHPELIRRIATEGHGLANHTMTHRDLSRCEPGEVRREISDANKVIRTVCPQACVHY 178
**Dg2*NodB2***' 16 EHAAKHPELIRRIAAEGHGLANHTMTHRDLSRCEPGEVRREISDANTIIRTVCPQACVHY 75
**Dg2*NodB3***’ 56 RHAAAHLEIISRMVASGHLLAGHSMTHRSLSRCDEQRTRWEIREARR-----APLTATRV 110

 * * * * * *
**Dg1*NodB1*** 114 LRTPYGRWNDGARRVAAELGLQPLGWTIDSRDWSAPGVTEIMDALRQQLHPGGIVLLHDG 173
**Dg2*NodB1*** 114 LRTPYGRWNDGARRVAAELGLQPLGWTIDSRDWSAPGVTEIMDALRQQLHPGGIVLLHDG 173
**Dg1*NodB2*** 179 LRAPYGAWT-GEARAAALFGLEPLNWSVDPRDWSRPGANVIVETVLTCIRPGGVILLRGG 237
**Dg2*NodB2***' 76 LQTPYSAWT-SEARAAALFGLEPLNWSV--------------------IRATG------- 107
**Dg2*NodB3***’ 111 LSPVFRCWR---------------RWP-------------------GRRHTDGIQLVR-- 134

 * * * * * *
**Dg1*NodB1*** 174 CLPERKR-GCSAGHQDQTLAVVPLLIRELRHQGLVPGPPLLEALPRLPAAGTPMGYGW 230
**Dg2*NodB1*** 174 CLPERKR-GCSAGHQDQTLAVVPLLIRELRHQGLVPGPPLLEALPRLPAAGTPMGYGW 230
**Dg1*NodB2*** 238 CPADEWPSGSRTGLRERTVIALRRLIPALHEYGFV-----FQPLPA-PSVHSPISRPV 289
**Dg2*NodB2***' 108 ------------------------------------------RVPA------------ 111
**Dg2*NodB3***’ 144 -----AP-ACEAAATDNQTAARTRSDRS-------------ESMPATDELLNEME--- 170

**C**

* * * * * *
**Dg1*nodC***  1 ATGTCGACCGCGGTGAGCATTCTCTCCACCGCCAACATGATCGTCCCCTCGTCCTATGCC 60
**Dg2*nodC***  1 ------------ATGAGCATTCTCTCCACCGCCAGCATGATCATACCGTCATCCTATGTC 48

 * * * * * *
**Dg1*nodC*** 61 ACCCTCGCCATTGGTTATCAGGGCGCGAAGACGTTTTATGCCCGGCGCGCGTACCGGCCG 120
**Dg2*nodC*** 49 ACCCTCGCCCTGGGCTATCAGGGCGCGAA**G**ACGTTTTATGCCCGGCGCG**C**TTTCCGGCCG 108

 * * * * * *
**Dg1*nodC*** 121 ACTACCACGCACCACGCAGACCCCTGCGACAGTCTGCCGAGTGTGGACGTGATTATCCCC 180
**Dg2*nodC*** 109 ACTACCATGTACCACGCAGACCCTTGCGACGTCCTGCCGAGTGCGGATGTGATTATT**C**CC 168

 * * * * * *
**Dg1*nodC*** 181 TGTTACCACGAGGACCCGCTGACCCTGGCAGCGTGCCTGCGTTCGGTCGCGGCACAGGAC 240
**Dg2*nodC*** 169 TGTTACCACGAGGACCCGCTGACCCTGGCAGCGTGCCTGCGTTCGGTCGCGGCCCAGGAC 228

 * * * * * *
**Dg1*nodC*** 241 TACCAGGGTGAGCTGCGGGTCTACCTCGTCGACGACGGCTCCACAAATCGCGACCGCCTG 300
**Dg2*nodC*** 229 TACCAGGGTGAGCTGCGGGTCTACGTCGTCGACGACGGCTCTAAAAATC**G**CGACCGCCTG 288

 * * * * * *
**Dg1*nodC*** 301 GAGCCCGTTTACGACACCTACGCCGGCGACGCGCGGTTCACCTTTCTGCTGCTCCCCCAC 360
**Dg2*nodC***  289 **G**GGCCTGTTTACGACACCTACGCCGGCGACCCGCGGTTCACCTTCCTGCTGCTCCCGCAC 348

 * * * * * *
**Dg1*nodC*** 361 AACGTCGGGAAGCGCAAGGCACAGGTCGCGGCGATCCGCCGGTCAGCCGGTGACCTGGTG 420
**Dg2*nodC*** 349 AACGTCGGGAAGCGCAAGGCACAGGTC**G**CGGCGATCCGCCGGTCAGCCGGTGACCTGGTG 408

 * * * * * *
**Dg1*nodC*** 421 GTCAACGTCGACTCGGACACCACCATCGAACCGGATGTCGTGCGTAAGTTGGCGGCGAAG 480
**Dg2*nodC*** 409 GTCAACGTCGACTCGGACACCATCATCGAACCGGATGTCGTGCGCAAGCTGGCGGCGAAG 468

 * * * * * *
**Dg1*nodC*** 481 ATGACCGATCCGGCCGTTGGAGCGGCCATGGGCCAGATGGTCGCCCGCAACCGACGTGCC 540
**Dg2*nodC*** 469 ATGACCGATCCGGCCGTGGGCGCGGCCATGGGCCAGATGGTCGCCCGCAACCGACGCGCC 528

 * * * * * *
**Dg1*nodC*** 541 ACCTGGCTGACCCGGCTGATCGATATGGAGTACTGGATCGCCTGTAATGAAGAGCGGGCG 600
**Dg2*nodC***  529 ACCTGGCTGACCCGATTGATCGATATGGAG**T**ACTGGATCGCCTGTAATGAGGAGCGGGCA 588

 * * * * * *
**Dg1*nodC*** 601 GCCCAGGCTGAGTTCGGCGCTGTCATGTGCTGCTGCGGGCCGTGCACGGTCTACCGTCGC 660
**Dg2*nodC*** 589 GCCCAGGCTGAGTTCGGCGCTGTCATGTGCTGCTGCGGGCCGTGCACGATCTACCGTCGC 648

 * * * * * *
**Dg1*nodC*** 661 TCCGTCCTCCTTCAGGTACTTGACCAGTACGAGACCCAGCTTTTCCGGGGGAGGCCGAGC 720
**Dg2*nodC*** 649 TCCGTCCTCCTTGCGGTGCTTGACCAGTACGAGACCCAGT**T**TTTCCGGGGGCGGCCGAGC 708

 * * * * * *
**Dg1*nodC*** 721 GACTTCGGTGAGGACCGTCACCTGACCATTCTCATGCTCAAGGCGGGCCTGCGTACCGAG 780
**Dg2*nodC*** 709 GACTTCGGTGAGGACCGCCACCTCACCATTCTCATGCTCAAGGCG**G**GCCTGCGTACCGAG 768

 * * * * * *
**Dg1*nodC*** 781 TACGTTCCGGACGCCACGGCGGCGACGGTGGTACCCGAACGACTTCGGCCATACCTGCGC 840
**Dg2*nodC*** 769 TACGTTCCGGACGCCACGGCGGCGACGGTAGTACCCGAGCGAATGCGGCCATACCTGAGC 828

 * * * * * *
**Dg1*nodC*** 841 CAGCAACTTCGGTGGGCGCGCAGCACATACCGCGATACGTTGCTGGCCATACGCCTGCTG 900
**Dg2*nodC*** 829 CAACAACTGCGGTGGGCGCGCAGCACATACCGCGAT**A**CGTTGCTGGC**C**ATACGCCTGTTG 888

 * * * * * *
**Dg1*nodC*** 901 CCCCGGCTCGGCCGCTATCTCATGCTGGACGTGGTAGGCCAGAACCTCGCACCGCTGTTG 960
**Dg2*nodC*** 889 CCCCGGCTCGGCCGCTATCT**C**ATGCTGGACGT**G**GTAGGCCAGAACCTCGCACCACTGTTG 948

* * * * * *
**Dg1*nodC*** 961 CTCGCCCTGACGGTGCTGACCGGATTCGCGCAGGTCGCGGCGACGGCGACAATACCGTGG 1020
**Dg2*nodC*** 849 CTCGGCCTGACGGTGCTGACCGGATTCGCGCAGATC**G**CGGCGACGGCGACGATACCGTGG 1008

 * * * * * *
**Dg1*nodC*** 1021 TGGCCGATCCTGGTGATCACGGCGGTGACCTTGATCAGTTGCTGCTGCGCGGCCTGGCAT 1080
**Dg2*nodC*** 1009 TG**G**CCGATCCTGGTGATCACGGCGGTGACCTTGATCAGTTGCTGCTGCGCGGCCTGGCAT 1068

 * * * * * *
**Dg1*nodC*** 1081 ACCCGCCAGGCGAGGTTTTTCGCCTTTGCGCTGCACACCTTTATCAATATCTTCCTGCTA 1140
**Dg2*nodC*** 1069 ACCCGCCAGGCGAGGTTTTTCGCCTTTACGCTGCACACCTTCATCAACATTTTCCTG**C**TA 1128

 * * * * * *
**Dg1*nodC*** 1141 CTCCCGCTGAAGGCCTACGCGATCTGTACCCTGAGCAACGCCAGCTGGGAGTCCCGCGTC 1200
**Dg2*nodC*** 1129 CTCCCGCTGAAAGCCTACGCGATCTGCACCCTGAGCAACGCCAACTGGGGTTCGCGCGCC 1188

 * * * * * *
**Dg1*nodC*** 1201 ATTCCGACCGCTGCCCCAGGTGGTTACGAAAAACGAGCGCCGACGGCCGTACTGCCCACG 1260
**Dg2*nodC*** 1189 ATTCCGGCCGCCGCCCCGGGAGGTTACGAAGAACGAGTGCCGACGGCCGCACTGCCCACG 1248

 * * * * * *
**Dg1*nodC*** 1261 CAGC------CCGTGCCTGCGCCTAATTCCGCCTCCACCACCTCCCCGCTGTCATCGTGA 1314
**Dg2*nodC*** 1249 CAACCCGGGACCGCGCCTGCGCCCGATTCCGCCCCCGCCACCTCCCCGCAGCCACGCTGA 1308

* * * * * *
**Dg1NodC**  1 MSTAVSILSTANMIVPSSYATLAIGYQGAKTFYARRAYRPTTTHHADPCDSLPSVDVIIP 60
**Dg2NodC** 1 ----MSILSTASMIIPSSYVTLALGYQGAKTFYARRAFRPTTMYHADPCDVLPSADVIIP 56

* * * * * *
**Dg1NodC**  61 CYHEDPLTLAACLRSVAAQDYQGELRVYLVDDGSTNRDRLEPVYDTYAGDARFTFLLLPH 120
**Dg2NodC**  57 CYHEDPLTLAACLRSVAAQDYQGELRVYVVDDGSKNRDRLGPVYDTYAGDPRFTFLLLPH 116

* * * * * *
**Dg1NodC** 121 NVGKRKAQVAAIRRSAGDLVVNVDSDTTIEPDVVRKLAAKMTDPAVGAAMGQMVARNRRA 180
**Dg2NodC** 117 NVGKRKAQVAAIRRSAGDLVVNVDSDTIIEPDVVRKLAAKMTDPAVGAAMGQMVARNRRA 176

* * * * * *
**Dg1NodC** 181 TWLTRLIDMEYWIACNEERAAQAEFGAVMCCCGPCTVYRRSVLLQVLDQYETQLFRGRPS 240

**Dg2NodC** 177 TWLTRLIDMEYWIACNEERAAQAEFGAVMCCCGPCTIYRRSVLLAVLDQYETQFFRGRPS 236

* * * * * *
**Dg1NodC** 241 DFGEDRHLTILMLKAGLRTEYVPDATAATVVPERLRPYLRQQLRWARSTYRDTLLAIRLL 300
**Dg2NodC** 239 DFGEDRHLTILMLKAGLRTEYVPDATAATVVPERMRPYLSQQLRWARSTYRDTLLAIRLL 296

* * * * * *
**Dg1NodC** 301 PRLGRYLMLDVVGQNLAPLLLALTVLTGFAQVAATATIPWWPILVITAVTLISCCCAAWH 360
**Dg2NodC** 297 PRLGRYLMLDVVGQNLAPLLLGLTVLTGFAQIAATATIPWWPILVITAVTLISCCCAAWH 356

* * * * * *
**Dg1NodC** 361 TRQARFFAFALHTFINIFLLLPLKAYAICTLSNASWESRVIPTAAPGGYEKRAPTAVLPT 420
**Dg2NodC** 357 TRQARFFAFTLHTFINIFLLLPLKAYAICTLSNANWGSRAIPAAAPGGYEERVPTAALPT 416

*
**Dg1NodC** 421 QP--VPAPNSASTTSPLSS 437

**Dg2NodC** 417 QPGTAPAPDSAPATSPQPR 435

**D**

* * * * * *
**Dg2*nodH1***  1 ATGAACCAGGAGAG**A**CGGGCGCTGTCGGCTCCAGAGCCATTTGTCATACTGGCAATGCCA 60
**Dg2*nodH2*** 1 ATGAACCAGGAGAGAGGGGCGCTGTTGACTCCAGAGCCATTTGTCATACTGACAATTCCA 60

 * * * * * *
**Dg2*nodH1*** 61 CGAACGGGATCACACCACCTGAAGGCGCTGGCCAACGAGCATCCGAACGTCTTGACCAAT 120
**Dg2*nodH2*** 61 CGAACGGGATCACACCACCTGGAGGCACTGGCCAACGAGCATCCGAACGTCTTGACCAAT 120

 * * * * * *
**Dg2*nodH1*** 121 GGAGA**G**ATACTTAATCCGTGGGACACCGACTGGCCCGGTGTCGATCGTACAGAAATGTCC 180
**Dg2*nodH2*** 121 GGAGAGCTACTTAATCCGTGGGACACCGGATGGCCTGGAGTCGATCGTACGGACATGTCC 180

 * * * * * *
**Dg2*nodH1*** 181 GATCGTGAACTGCTCGAGCTGGCCTTCGTGCGCTTTCCGCGGCGAGATGAAAAATACGAG 240
**Dg2*nodH2*** 181 GACGGGGAACTGCTCGAGCTGGCCTTCGTGCGTTTTCCGAGGCGAGACGAAAAATACGAG 240

 * * * * * *
**Dg2*nodH1*** 241 GTCAGTTGCGTCGGCTGCAAGGTCAATGAACCTCAGTTCCACGAACG**T**CCCA**C**CTT**T**TTT 300
**Dg2*nodH2*** 241 GTCAGTTGCGTCGGCTGCAAGATCAATGAACCTCAGTTCCACGAACGTCCCACCTTTTTT 300

 * * * * * *
**Dg2*nodH1*** 301 GACGAGCTGGCGGCTTGGCCGCGCCTGAAAGTAATCGCGCTCCAACGACGGAACCTGCTC 360
**Dg2*nodH2*** 301 GAAGAGCTGGCGGCGTGGCCGCGTCTGAAAGTAATCGTGCTCCAAAGGCGGAACCTGCTC 360

 * * * * * *
**Dg2*nodH1*** 361 GAGTCATTCCGGTCCTTCATCCAGG**C**GCG**T**GAGAGCGGCCGCTGGCTGGCCCCCAGTGCG 420
**Dg2*nodH2*** 361 GAATCGTTCCGGTCCCTCACCCAGGCGCGCGACAGTGGCCGCTGGCTGGCCCCCAGCGCG 420

 * * * * * *
**Dg2*nodH1*** 421 GACGGCCCGGCGCCGGTGCCGCCGCGGGT**G**AAGCTGTCCCCCGCAGGCTGCGAGAGTTAC 480
**Dg2*nodH2*** 421 GACGGCCCGACGCCAGTGCCGCCGCGGGTGAAGCTGTCGCCCGTGGACTGCGAGAGCTAC 480

 * * * * * *
**Dg2*nodH1*** 481 TTCAGAAGCGCCGAGGA**A**TTCTACGGTCGCATCTTCGCCCGTTTTTCCCCGGAGAAGATC 540
**Dg2*nodH2*** 481 TTCAGAAGCGCAGAGCGCTTCTACGGCCGCATCTTCGCCCGTTTTCCTCCGGAGAGGATC 540

 * * * * * *
**Dg2*nodH1*** 541 CACGA**A**ATT**T**ACTATGAGGACCTGCGGGACAGACCGGGGGAATGCATGGCGGGGATC**T**GG 600
**Dg2*nodH2*** 541 CACGGAATTTACTATGAGGACCTGGGGGACAGCCCGAGGGAATGCCTGGCGAGGATCTGG 600

 * * * * * *
**Dg2*nodH1*** 601 GACTTTCTGGGCGTTTCCCCGCATCTGCTGTCCGGCTGTCTTCTCCTGCAACAGCAGGAG 660
**Dg2*nodH2*** 601 GATTTTCTCCGCGTTCCCCCGCATCCGCTGTCCGACTGTCGTCTCCTGCAACGGCAGGAG 660

 * * * * * *
**Dg2*nodH1*** 661 ACACGCCCGCTCAGCGA**G**GCGGTGCTGAACTACGCCGAGCTACG**C**GGCCACTTCCGGGGC 720
**Dg2*nodH2*** 661 ACGCGGCCGCTCGGCGAAGCGGTGCTAAACTATGCCGAGCTGCGCGACCACTTCCGGGGC 720

 * *
**Dg2*nodH1*** 721 ACTCCGTACC**A**GGCTTTCTTTGAATGA 747
**Dg2*nodH2*** 721 ACCCCGTACCAGGCTTTCTTTGAATGA 747

* * * * * *
**Dg2NodH1** 1 MNQERRALSAPEPFVILAMPRTGSHHLKALANEHPNVLTNGEILNPWDTDWPGVDRTEMS 60
**Dg2NodH2**  1 MNQERGALLTPEPFVILTIPRTGSHHLEALANEHPNVLTNGELLNPWDTGWPGVDRTDMS 60

 * * * * * *
**Dg2NodH1** 61 DRELLELAFVRFPRRDEKYEVSCVGCKVNEPQFHERPTFFDELAAWPRLKVIALQRRNLL 120
**Dg2NodH2** 61 DGELLELAFVRFPRRDEKYEVSCVGCKINEPQFHERPTFFEELAAWPRLKVIVLQRRNLL 120

 * * * * * *
**Dg2NodH1** 121 ESFRSFIQARESGRWLAPSADGPAPVPPRVKLSPAGCESYFRSAEEFYGRIFARFSPEKI 180
**Dg2NodH2** 121 ESFRSLTQARDSGRWLAPSADGPTPVPPRVKLSPVDCESYFRSAERFYGRIFARFPPERI 180

 * * * * * *
**Dg2NodH1** 181 HEIYYEDLRDRPGECMAGIWDFLGVSPHLLSGCLLLQQQETRPLSEAVLNYAELRGHFRG 240
**Dg2NodH2** 181 HGIYYEDLGDSPRECLARIWDFLRVPPHPLSDCRLLQRQETRPLGEAVLNYAELRDHFRG 240


**Dg2NodH1** 241 TPYQAFFE 248
**Dg2NodH2** 241 TPYQAFFE 248

**E**

* * * * * *
**Dg1*nltI*** 1 ATGGATAATGGGATCCACACCGAGAATCTGACCAAGAAGTACGGGGCGGTGTACGGCCTG 60
**Dg2*nltI*** 1 ATGGATAATGGGATCCACACCGAGAATCTGACCAAGAAGTACGGGGCGGTGTACGGCCTG 60

 * * * * * *
**Dg1*nltI*** 61 CACGGCCTGAACCTGCAGGTTTCGGCCGGGAGCGTACTCGGGCTGCTGGGCCCGAACGGG 120
**Dg2*nltI*** 61 CACGGTTTGAACCTGCAGGTTTCGGCCGG**G**AGCGTACT**C**GGACTGCTGGGCCCGAACGGG 120

 * * * * * *
**Dg1*nltI*** 121 GCCGGCAAGACCACGACTGTGAACATACTGACGACGCTTTTGAAACCGGACGGTGGCAGT 180
**Dg2*nltI*** 121 GCCGGCAAGACCACGACTGTGAACATACTGACGAC**G**CTTTTGAAGCCGGACGGTGGCAGT 180

 * * * * * *
**Dg1*nltI*** 181 GCCTGGGTGGGGGGCTTCAACGTGGCCCGCCACCCACTGCAGGTACGGCGCCGGATCGGC 240
**Dg2*nltI*** 181 GCCTGGGTGGGGGGCTTCAACGTGGCCCGCCACCCACTGCAGGTACGGCGCCGGATCGGC 240

 * * * * * *
**Dg1*nltI*** 241 GTCTCCGGCCAGGAAACCGCTGTGGAGCCCCTGCTTACCGGCGCGGAAAACCTGGAGCTG 300
**Dg2*nltI*** 241 GTCTCCGGCCAGGAAACCGCTGTGGAGCCCCTGC**T**TACCGGTGCGGAGAACCTGGAGCTG 300

 * * * * * *
**Dg1*nltI*** 301 TTCGGCCGGCTGCATCGCCTCAGCCGGCGGCATGCCCGCGGGCGGGCCCAGGAGCTGCTG 360
**Dg2*nltI*** 301 TTCGGCCGGCTGCATCGCCTCAGCCGGCGGCATGCCCGCGGGCG**G**GCCCAGGAGCTGCTG 360

 * * * * * *
**Dg1*nltI*** 361 GAAATGTTCGATCTCACCGCGGCGGCCGGGCGACTCGCACGAACCTACTCGGGTGGCATG 420
**Dg2*nltI*** 361 GAAATG**T**TC**G**ATCTCACCGCGGCGGCCGGGCGACTCGCACGAACCTACTCGGGTGGCATG 420

 * * * * * *
**Dg1*nltI*** 421 AGACGCCGCCTCGACCTCGCCATCAGTTTGATCAAGCGACCCTCGATTCTGTTCCTGGAC 480
**Dg2*nltI*** 421 AGACGCCGCCTCGACCTCGCCATCAGTTTGATCAAGCGACCCTCGA**T**TCTGTTCCTGGAC 480

 * * * * * *
**Dg1*nltI*** 481 GAACCCACCACCGGACTCGACCCGCGCAGTCGCACTGCCACCTGGGATCTCATCAGGGAG 540
**Dg2*nltI*** 481 GAACCCACCACCGGACTCGA**C**CCGCGCAGTCGCACCGCCACCTGGGATCT**C**ATCAGGGAG 540

 * * * * * *
**Dg1*nltI*** 541 CTTGTCGGCTCCGGCGTGACGCTCCTGCTCACCACCCAGTACCTGGAGGAAGCGGACCAG 600
**Dg2*nltI*** 541 CTTGTCAGCTCCGGCGTGACGCTCCTGCTCACCACCCAGTACCTGGAGGAAGCGGACCAG 600

 * * * * * *
**Dg1*nltI*** 601 CTCGCCGACCTGATCGCCGTGGTGGACCAGGGCTGCCTCATCGCCGAAGGCACCGTGGAA 660
**Dg2*nltI*** 601 CTCGCCGACCTGATCGCAGTGGTGGACCAGGGCCGCCTCATCGCCGAAGGCACCGTGGAA 660

 * * * * * *
**Dg1*nltI*** 661 GAACTGAAGACCCGAACCAGCGACGACCGCATCGAGATCGTCCTGCGCGACCATGAGCTG 720
**Dg2*nltI*** 661 GAACTGAAGACCCGAACCAGCGACGACCGCATCGAGATCGTCCTGCGCGACCATGAGCTG 720

* * * * * *
**Dg1*nltI*** 721 CTGCCTCTGGCCGCGACCATCCTCGACCGTTTCGCAACGTCGGAGGCTGTCGTCACGCGA 780
**Dg2*nltI*** 721 CTGCCTCTGGCCGCGACCATCCTCGACCGTTTCGCGACGTCGGAGGCTGTCGTCACGCGA 780

 * * * * * *
**Dg1*nltI*** 781 TCGGAACGGCGGATTGCCGTCATGGCACCGCACAAACCAGGCCTGCTGACGCAGGTGCTG 840
**Dg2*nltI*** 781 TCGGAACGGCGGATTGCCGTCATGGCACCGCACAAACCAGGCCTGCTGACTCAGGTGCTG 840

 * * * * * *
**Dg1*nltI*** 841 CGCGAGATGGACGTCGCGGCGATCGAGCTCGAGGACGTCGAGTTGCGCCGGCCGACCCTC 900
**Dg2*nltI*** 841 CGCGAGATGGACGCCGCGGCGATCGAGCTCGAGGACGTCGAGTTGCGCCGGCCGACCCTC 900

 * * * * * *
**Dg1*nltI*** 901 GACGACGTCTTCTTTACCTTGACGGGGCGCCCCATGGACGTCAAGGCTCAGCACGAGATG 960
**Dg2*nltI*** 901 GACGACGTCTTCTTCACCTTGACGGGGCACCCCATGGACGTCAAGGCTCAGCGCGAGATG 960

 *
**Dg1*nltI*** 961 GCTACGGTGGAATGA 975
**Dg2*nltI*** 961 GCTACGGTGGAATGA 975

* * * * * *
**Dg1NltI** 1 MDNGIHTENLTKKYGAVYGLHGLNLQVSAGSVLGLLGPNGAGKTTTVNILTTLLKPDGGS 60
**Dg2NltI** 1 MDNGIHTENLTKKYGAVYGLHGLNLQVSAGSVLGLLGPNGAGKTTTVNILTTLLKPDGGS 60

* * * * * *
**Dg1NltI** 61 AWVGGFNVARHPLQVRRRIGVSGQETAVEPLLTGAENLELFGRLHRLSRRHARGRAQELL 120
**Dg2NltI** 61 AWVGGFNVARHPLQVRRRIGVSGQETAVEPLLTGAENLELFGRLHRLSRRHARGRAQELL 120

 * * * * * *
**Dg1NltI** 121 EMFDLTAAAGRLARTYSGGMRRRLDLAISLIKRPSILFLDEPTTGLDPRSRTATWDLIRE 180
**Dg2NltI** 121 EMFDLTAAAGRLARTYSGGMRRRLDLAISLIKRPSILFLDEPTTGLDPRSRTATWDLIRE 180

 * * * * * *
**Dg1NltI** 181 LVGSGVTLLLTTQYLEEADQLADLIAVVDQGCLIAEGTVEELKTRTSDDRIEIVLRDHEL 240
**Dg2NltI** 181 LVSSGVTLLLTTQYLEEADQLADLIAVVDQGRLIAEGTVEELKTRTSDDRIEIVLRDHEL 240

 * * * * * *
**Dg1NltI** 241 LPLAATILDRFATSEAVVTRSERRIAVMAPHKPGLLTQVLREMDVAAIELEDVELRRPTL 300
**Dg2NltI** 241 LPLAATILDRFATSEAVVTRSERRIAVMAPHKPGLLTQVLREMDAAAIELEDVELRRPTL 300

 * *
**Dg1NltI** 301 DDVFFTLTGRPMDVKAQHEMATVE 324
**Dg2NltI** 301 DDVFFTLTGHPMDVKAQREMATVE 324

**F**

* * * * * *
**Dg1*nltJ*** 1 ATGAAGCCGAATACAATCATCGACGGCGCCGCCGCATCGAACGGTGAGAAGCAGGTCGTC 60
**Dg2*nltJ*** 1 ATGAAGCCGAATACAATCATCGACGGCGCCGCCGCAT**C**GGACGGTGAGAAGCAGGTCGTC 60

 * * * * * *
**Dg1*nltJ*** 61 GCCGGTCGTCATCGGAGCACGCGGTCACATCACGTCACCCGCGGCTGGGCCGGGCCAGGG 120
**Dg2*nltJ*** 61 GCCGGTCGTCATCGGAGCACGCGGTC**A**CATCACGTCACCCGCAGCTGGGCCGGGCCAGGG 120

 * * * * * *
**Dg1*nltJ*** 121 AAACCGGAGGGGATCATCCCTGTCACGTTCCTCATCGATGTCTACCTGTTGACCAGGCGT 180
**Dg2*nltJ*** 121 AAACTGGAGAGGATCACCCCTGTCACGTTCCTCATCGATGTCTACCTGTTGACCAGGCGT 180

 * * * * * *
**Dg1*nltJ*** 181 CTGCTGATGCGCATGCCGCACCAGCCCGACCTCATCGTCTACAGCATCGTCCAACCAGCC 240
**Dg2*nltJ*** 181 CTGCTG**A**TGCGCATGCCGCACCAGCCCGACCTCATCGTCTACAGCATCGTCCAACCAGCC 240

 * * * * * *
**Dg1*nltJ*** 241 ATCTTCACCTTAGCGATGATATATCTCTTCGGCAAGGCCATCACACTTCCCGGCGAGGCC 300
**Dg2*nltJ*** 241 ATTTTCACCTTAGCGATGATATATCTTTTCGGCAAGGCCATCGCACTTCCCGGCGGG**G**CC 300

 * * * * * *
**Dg1*nltJ*** 301 GACTACACGGATTTCGTCATCGCTGGGCTGCTGGCCCAAACGGTCGTCATGGGCGCCGCC 360
**Dg2*nltJ*** 301 AATTACACGGATTTCGTCATCGCTGGACTGCTGGCCCAGAC**G**GTCGTCATGGGCGCCGCC 360

 * * * * * *
**Dg1*nltJ*** 361 TCGGCCACGTCCGGCGGAATCGCCTTCGAACTATCCCAGAAGACGATCGACCGGCTCCGT 420
**Dg2*nltJ*** 361 TC**G**GCCAC**G**TCCGGTGGAATCGCCTTCGAACTATCCCAGAAGACGATCGATCGGCTCCGT 420

 * * * * * *
**Dg1*nltJ*** 421 ACGCTCCCCCTGTCCCGGCTAGCCATCCTCGCGGGTTGCACCAATGCCGGCGTCATAAGG 480
**Dg2*nltJ*** 421 ACGCTCCCCCTGTCTCGGCT**G**AGTATCCTCGCGGGTTGCACCAATGCCGGCGTCATAAGG 480

 * * * * * *
**Dg1*nltJ*** 481 GCCTTCATCACGGTGTTCGTGACGACAGTGTGTGGGCTGGTGGCCGGGTGGCGTGTGCAG 540
**Dg2*nltJ*** 481 GCCTTCATCACGGTGTTCGTGACGACGGTGTGTGGGCTGGTGGCCGGGTGGCGGGCACAG 540

 * * * * * *
**Dg1*nltJ*** 541 GCCGGGCTGGGCAACGCGCTCCTCGCATTTCTTATTCTGCTGCTGTTCGGTCTGGCAATG 600
**Dg2*nltJ*** 541 GCCGGGCTGGGCGGTGCGCTCCTCGCGTTTCTTATTCTGCTGCTGTTCGGTCTGGCAATG 600

 * * * * * *
**Dg1*nltJ*** 601 AGCTGGGTGGGAGCGCTGATCGGAGTGTCGGTCAGCAATCCCGAAGTGGCCGCCGGAGCG 660
**Dg2*nltJ*** 601 AGCTGGGTGGGAGCGCTGATCGGAGTGTCGGTCAGCAATCCCGAAGTGGCCGCCGGAGCG 660

 * * * * * *
**Dg1*nltJ*** 661 GGTACCGTCTGGCTTTTTCCGGTCATGTATCTGTCCAATGCGCTCACCCCGATGAGCGCC 720
**Dg2*nltJ*** 661 GGTACCGTCTGGCTTTTTCCGGTCATGTATCTGTCCAATGCGCTCACCCCGGTGAGCGCC 720

 * * * * * *
**Dg1*nltJ*** 721 ATGCCAGGCTGGCTCCAGCCCGTCGCCGGGTGGAACCCGTTGTCCGCTGTCACGACTGCC 780
**Dg2*nltJ*** 721 ATGCCGGGCTGGCTCCAGCCCGTCGCCGGGTGGAACCCGTTGTCCGCTGTCACGACTGCC 780

 * * * * * *
**Dg1*nltJ*** 781 TGTCGTCAGTTGTTCGGAAATCCGACGGCGTCCGGTGCCGAGAATATCTGGCCGGCTGAT 840
**Dg2*nltJ*** 781 TGTCGTCAGTTGTTCGGAAATCCGACGGCGTTCGGTGTCGAGAATATCTGGCCGGCGGAT 840

 * * * * * *
**Dg1*nltJ*** 841 CATCCGATCCTGGCATCGGTTTCGTGGTCACTGGCGATAATGCTGGCCACCGTGCCGCTG 900
**Dg2*nltJ*** 841 CATCCGATCCTGGCATCGGTTTCGTGGTCACTGGCGATAATGCTTGTCACCGTGCCGCTG 900

 * * *
**Dg1*nltJ*** 901 ACCGTTTGGAAATTCGTGCGGCGGACAAGCAGATGA 936
**Dg2*nltJ*** 901 ACCGTTTGGAAATTCGTGCGGTGGACAAGCAGATGA 936

* * * * * *
**Dg1NltJ**  1 MKPNTIIDGAAASNGEKQVVAGRHRSTRSHHVTRGWAGPGKPEGIIPVTFLIDVYLLTRR 60
**Dg2NltJ**  1 MKPNTIIDGAAASDGEKQVVAGRHRSTRSHHVTRSWAGPGKLERITPVTFLIDVYLLTRR 60

* * * * * *
**Dg1NltJ**  61 LLMRMPHQPDLIVYSIVQPAIFTLAMIYLFGKAITLPGEADYTDFVIAGLLAQTVVMGAA 120
**Dg2NltJ** 61 LLMRMPHQPDLIVYSIVQPAIFTLAMIYLFGKAIALPGGANYTDFVIAGLLAQTVVMGAA 120

 * * * * * *
**Dg1NltJ** 121 SATSGGIAFELSQKTIDRLRTLPLSRLAILAGCTNAGVIRAFITVFVTTVCGLVAGWRVQ 180
**Dg2NltJ** 121 SATSGGIAFELSQKTIDRLRTLPLSRLSILAGCTNAGVIRAFITVFVTTVCGLVAGWRAQ 180

 * * * * * *
**Dg1NltJ** 181 AGLGNALLAFLILLLFGLAMSWVGALIGVSVSNPEVAAGAGTVWLFPVMYLSNALTPMSA 240
**Dg2NltJ** 181 AGLGGALLAFLILLLFGLAMSWVGALIGVSVSNPEVAAGAGTVWLFPVMYLSNALTPVSA 240

 * * * * * *
**Dg1NltJ** 241 MPGWLQPVAGWNPLSAVTTACRQLFGNPTASGAENIWPADHPILASVSWSLAIMLATVPL 300
**Dg2NltJ** 241 MPGWLQPVAGWNPLSAVTTACRQLFGNPTAFGVENIWPADHPILASVSWSLAIMLVTVPL 300

 *
**Dg1NltJ** 301 TVWKFVRRTSR 311
**Dg2NltJ** 301 TVWKFVRWTSR 311

**Figure S2.** **Alignments of *nod* gene and Nod protein sequences (including *nltIJ*/NltIJ) from cluster II *Frankia* strains Dg1 and Dg2.** In the nucleotide sequences of non-truncated genes, the SNP positions (ca. 50% SNPs, i.e., Dg2a/Dg2b differences) in the Dg2 metagenome are highlighted in **red**. **(A)** *nodA/*NodA, **(B)** *nodB/*NodB, **(C)** nodC/NodC, **(D)** NodH, **(E)** *nltI/*NltI, **(F)** *nltJ/*NltJ. Nucleotide and amino acid sequence alignments were performed using MUSCLE (**mu**ltiple **s**equence **c**omparison by **l**og- **e**xpectation; Edgar 2004) at the EMBL-EBI website. For *nodA* and *nodB*, some parts of the alignment were adjusted manually.

**List of SNP positions:**

Dg2*nodA*: 11, 67, 197, 271, 395, 499

Dg2*nodB1*: 56, 91, 256, 367, 444, 445, 446, 511, 637

Dg2*nodB2*: 129, 237, 365, 375, 421, 566, 567, 601

Dg2*nodB3’*: 23, 153, 189, 211, 290, 388, 478 (not labeled since gene is truncated)

Dg2*nodC*: 78, 98, 166, 278, 289, 376, 559, 689, 754, 865, 876, 921, 985, 1011, 1126

Dg2*nodH1*: 15, 78, 126, 288, 293, 297, 386, 390, 450, 498, 546, 550, 598, 678, 705, 731

Dg2*nodH2*: no SNPs

Dg2*nltI*: 90, 99, 156, 275, 345, 367, 370, 467, 501, 531

Dg2*nltJ*: 38, 87, 178, 298, 342, 363, 369, 441
