# Supplementary material for: An assemblage of Frankia Cluster II strains from California contains the canonical nod genes and also the sulfotransferase gene nodH
Source: BMC Genomics. 2016 Oct 12;17:796. doi: 10.1186/s12864-016-3140-1 (PMC5059922; doi:10.1186/s12864-016-3140-1)
Supplement: Additional file 4: — Candidatus Frankia datiscae Dg2-induced nodules on roots of D. glomerata and D. cannabina. (PPTX 5298 kb) [file 12864_2016_3140_MOESM4_ESM.pptx]

## Slide 1
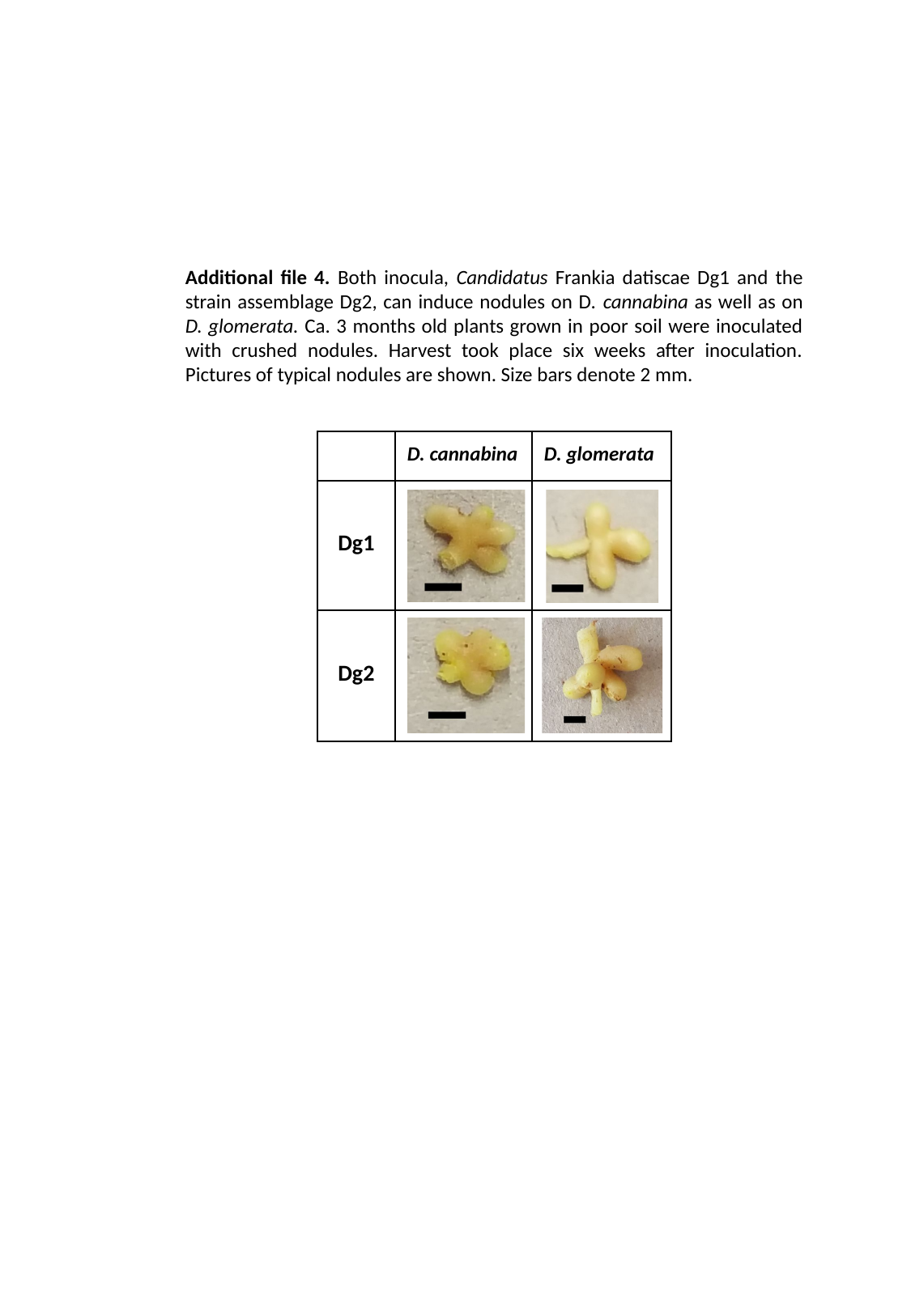

Additional file 4. Both inocula, Candidatus Frankia datiscae Dg1 and the strain assemblage Dg2, can induce nodules on D. cannabina as well as on D. glomerata. Ca. 3 months old plants grown in poor soil were inoculated with crushed nodules. Harvest took place six weeks after inoculation. Pictures of typical nodules are shown. Size bars denote 2 mm.
| | D. cannabina | D. glomerata |
| --- | --- | --- |
| Dg1 | | |
| Dg2 | | |
