# Supplementary material for: An assemblage of Frankia Cluster II strains from California contains the canonical nod genes and also the sulfotransferase gene nodH
Source: BMC Genomics. 2016 Oct 12;17:796. doi: 10.1186/s12864-016-3140-1 (PMC5059922; doi:10.1186/s12864-016-3140-1)
Supplement: Additional file 5: — Expression of nod genes in nodules induced by Dg2 on roots of D. glomerata. (DOCX 13 kb) [file 12864_2016_3140_MOESM5_ESM.docx]

**Additional file 5.** Expression of *nod* genes in nodules induced by Dg2 on roots of *D. glomerata* as compared to expression of the translation initiation factor gene *IF-3.* No amplification took place with *nodH1* or *nodH2* primers.

Expression of the Dg2 genes *nodA, nodB1* and *nodC* in *D. glomerata* nodules as tested by RT-qPCR.

| **Gene** | **Relative expression level compared to *IF-3*** |
| --- | --- |
| *nodA* | 4.58 ± 0.89 |
| *nodB1* | 6.65 ± 2.38 |
| *nodC* | 1.50 ± 0.51 |

The expression values are given in relative units and represent means ± standard deviation (n=3).

**List of primers used for qPCR experiment.**

| **Gene** | **Forward primer** | **Reverse primer** |
| --- | --- | --- |
| *IF-3* | GGATCAGAGTACCCGAGGTG | CCGTCTGGACCTGGTTCTTA |
| *nodA* | GTGCTGCGTCGTTTTATCAG | CCGGATTGATAACCGATTCC |
| *nodB1* | CGGACCGGATCCTTATTACA | CCATTATTTCCCACCGTGTC |
| *nodC* | GGATCGCCTGTAATGAGGAG | GCCTTGAGCATGAGAATGGT |
| *nodH1* | GCAAGGTCAATGAACCTCAG | GTTGGAGCGCGATTACTTTC |
| *nodH1* | AATGTCCGATCGTGAACTGC | CGTGGAACTGAGGTTCATTG |
| *nodH2* | GGCGAGACGAAAAATACGAG | AACGATTCGAGCAGGTTCC |
| *nodH2* | TGACTCCAGAGCCATTTGTC | CTCCATTGGTCAAGACGTTC |
| *nodH2* | GAGGCGAGACGAAAAATACG | AACGATTCGAGCAGGTTCC |
| *nodH2* | CGAGGATCTGGGATTTTCTC | CATAGTTTAGCACCGCTTCG |

Primer design took place using **primer3plus.**

Untergasser A, Nijveen H, Rao X, Bisseling T, Geurts R Leunissen J. Primer3Plus, an enhanced web interface to Primer3. Nucleic Acids Research 2007; 35: W71-W74; doi:10.1093/nar/gkm306
